# Natural Products as Anticancer Agents: Current Status and Future Perspectives

**DOI:** 10.3390/molecules27238367

**Published:** 2022-11-30

**Authors:** Abid Naeem, Pengyi Hu, Ming Yang, Jing Zhang, Yali Liu, Weifeng Zhu, Qin Zheng

**Affiliations:** 1 Key Laboratory of Modern Preparation of Chinese Medicine, Ministry of Education, Jiangxi University of Chinese Medicine, Nanchang 330004, China; 2 Key Laboratory of Pharmacodynamics and Safety Evaluation, Health Commission of Jiangxi Province, Nanchang Medical College, Nanchang 330006, China; 3 Key Laboratory of Pharmacodynamics and Quality Evaluation on Anti-Inflammatory Chinese Herbs, Jiangxi Administration of Traditional Chinese Medicine, Nanchang Medical College, Nanchang 330006, China

**Keywords:** natural products, bioactive anti-tumor agents, phytochemicals, anti-cancer plants, chemotherapy, chemoprevention

## Abstract

Natural products have been an invaluable and useful source of anticancer agents over the years. Several compounds have been synthesized from natural products by modifying their structures or by using naturally occurring compounds as building blocks in the synthesis of these compounds for various purposes in different fields, such as biology, medicine, and engineering. Multiple modern and costly treatments have been applied to combat cancer and limit its lethality, but the results are not significantly refreshing. Natural products, which are a significant source of new therapeutic drugs, are currently being investigated as potential cytotoxic agents and have shown a positive trend in preclinical research and have prompted numerous innovative strategies in order to combat cancer and expedite the clinical research. Natural products are becoming increasingly important for drug discovery due to their high molecular diversity and novel biofunctionality. Furthermore, natural products can provide superior efficacy and safety due to their unique molecular properties. The objective of the current review is to provide an overview of the emergence of natural products for the treatment and prevention of cancer, such as chemosensitizers, immunotherapeutics, combinatorial therapies with other anticancer drugs, novel formulations of natural products, and the molecular mechanisms underlying their anticancer properties.

## 1. Introduction

Noncommunicable diseases (NCDs) refer to diseases that are not transmitted directly from one individual to another, and account for more than 70% of all deaths worldwide (41 million deaths per year). Cancer is the second leading cause of death among NCDs, after cardiovascular disease [1]. The incidence and mortality of cancer are rapidly increasing throughout the world due to an increase in the aging population. The reasons are complex, including environmental pollution, chemical toxins pollution, ionizing radiation, free radical toxin, microorganisms (bacteria, fungi, viruses, etc.) and their metabolic toxins, genetic characteristics, endocrine imbalance, and immune dysfunction, etc. [2]. Global Cancer Observatory (GLOBOCAN) estimates that 19 million new cancer cases will be reported and 10 million deaths will be caused by this disease in 2020. Cancers in both sexes are most commonly diagnosed in the breast, lung, colon, prostate, and stomach (excluding non-melanoma skin cancer). It is estimated that by 2040, the incidence of cancer in the world population will increase to 30.2 million cases and the mortality rate will increase to 16.3 million cases, respectively [3].

The term “cancer” is used to refer to a large group of diseases (over 277) which are characterized by the uncontrolled growth of abnormal cells and which are caused by a wide range of factors [4]. In fact, any cell in the body is capable of becoming a cancer cell (carcinogenesis) once it has been subjected to a series of successive gene mutations. Specifically, this process occurs in three stages, namely: (a) the initiation stage, when the alteration has already been identified, (b) the promotion stage, where the altered cells are considered totally mutated and then the malignancy of the cells commences, and (c) the progression stage, once the tumor has already grown, and as the cells begin to divide in an accelerated and irreversible manner. Cancer cells may also spread to other parts of the body (metastasis) in addition to growing locally, which is an often cited cause of death from the disease [5].

Cancer research has always been a challenge because of its complexity. Different types of cancer may exhibit substantial differences in relation to genetic alterations, organ involvement, prognosis, and therapeutic management [6]. Even though various treatment options are available, their success relies upon the type and phase of the disease. Among various treatment alternatives, careful surgical removal of malignant tissues/tumor, radiation treatment, chemotherapy, and immunotherapy are generally employed. The effects of surgery and radiotherapy are local, while those of chemotherapy and targeted therapy are systemic. The type and stage of cancer determine whether such therapies are used alone or in combination with other treatments (e.g., radiotherapy and chemotherapy) [7]. Small molecule targeted therapy and chemotherapy are two therapeutic approaches used to treat cancer using chemical compounds. In general, chemotherapy drugs act as cytotoxic agents that interfere with various phases of the cell cycle. The rationale for their application is that cancer cells generally have a faster division rate than normal cells, making them more susceptible to chemotherapeutic agents [1]. Generally, these drugs can be classified into five categories according to their biochemical properties: alkylating agents (for example, cisplatin), antimetabolites (5-fluoroacil), antitumor antibiotics (doxorubicin), topoisomerase inhibitors (topotecan), and tubulin-binding drugs (paclitaxel) [8].

Despite the effectiveness of chemotherapeutic drugs, they can also cause adverse reactions in normal cells, including nausea, vomiting, mucositis, alopecia, neuropathy, alopecia, and myelosuppression. Furthermore, they have been found to be associated with multidrug resistance (MDR), an undesirable phenomenon responsible for more than 90% of the deaths of cancer patients undergoing chemotherapy [9]. Small molecule targeted therapy (SMTT) differs from chemotherapy in that it uses chemicals that target specific molecular targets within cancer cells. It is thought that these targets are modified genetically in cancer and are essential to the development and survival of tumors. The majority of the time, they are implicated in signaling pathways that are dysregulated during cancerous development [10]. The use of target-oriented compounds in clinics includes tyrosine kinases, proteasomes, and poly ADP-ribose polymerase inhibitors such as imatinib, carfilzomib, and ribociclib. The specificity of SMTT drugs is expected to result in a less toxic effect on healthy cells. However, side effects (such as a rash, diarrhea, or hypertension) have been reported [11]. Moreover, they may also trigger mechanisms that lead to the development of drug resistance. Even though these restorative choices are effective in treating different sorts of malignant growths, they have impediments; for example, recurrence of cancer, noncompliance because of extremely unfavorable implications such as fatigue, pain, nausea, anemia, emesis, and baldness, among the symptoms endured by patients. It is important to note that the vast majority of synthetic chemotherapeutic drugs developed before failed to satisfy the needs during clinical trials, despite the higher cost of expenditure. Thus, efforts continue to be made to find better alternatives that balance efficacy and toxicity and comply with drug resistance prevention measures.

The heterogeneous nature of cancer has limited the efficacy of conventional therapies such as radiation and standard chemotherapy in treating and preventing it, as they are likely to kill both normal and cancerous cells in the process, resulting in serious hematological toxicities and damage to the tissues involved [12]. A growing number of patients acquire or develop multiple drug resistance, making chemotherapy a treatment with limited therapeutic benefits. A number of anticancer drugs have also been associated with significant side effects, including cardiotoxicity caused by doxorubicin [13], ototoxicity caused by cisplatin, and cognitive impairment caused by 5-fluorouracil [14]. The adverse effects of chemotherapy on patients, such as kidney damage, gastrointestinal problems, hair loss, and fatigue, compromise adherence to treatment. A negative perception of treatment is also a result of these factors [15].

The majority of the chemotherapeutic drugs used in the clinic today are directed at only one particular target such as specific nucleic acids, particular proteins, or tumorigenic pathways. Platinum drugs, such as oxaliplatin, carboplatin, and cisplatin, are known to inhibit nucleotide synthesis and metabolism along with damaging DNA. Tyrosine kinase inhibitors such as gefitinib, erlotinib, and icotinib are directed at tyrosine kinase. Angiogenesis is inhibited by bevacizumab, sunitinib, and sorafenib [16]. The treatment of cancer has been greatly improved by the development of many drugs, but the disease is still an evolutionary process. Cancer cells are capable of adapting to a given drug treatment. Natural products have been observed to influence multiple oncogenic signaling pathways simultaneously by modulating the activity or expression of their molecular targets. Various natural products affect multiple pathways, including apoptotic cell death, cell proliferation, migration/invasion, angiogenesis, and metastasis. Natural products are capable of generating intracellular signals that trigger events that lead to the death of cancer cells. Chemotherapy can be enhanced by natural products in cases where cancer is resistant to treatment. The high cost of conventional drugs and the rising incidence of cancer have challenged researchers to find more cost-effective and eco-friendly alternatives. In this context, natural products are highly advantageous due to their chemical diversity, low toxicity, safety, and availability, which make them an attractive and affordable alternative to synthetic products [17,18].

In this review, we summarize novel studies and viewpoints on cancer therapy and natural products mostly from 2012 to 2022 (older sources were also cited if necessary) using Web of Science (http://www.webofknowledge.com, accessed on 1 July 2022), Google Scholar (https://scholar.google.com, accessed on 1 April 2022), PubMed (https://www.ncbi.nlm.nih.gov/pubmed, accessed on 1 June 2022), ScienceDirect (https://www.sciencedirect.com, accessed on 1 May 2022), Chinese National Knowledge Infrastructure (CNKI, https://www.cnki.net, accessed on 1 March 2022), Scopus (https://www.scopus.com, accessed on 1 April 2022), and Clinical Trials (https://clinicaltrials.gov, accessed on 1 November 2021). This study provides important insights into the efficacy and mechanism of action of natural products in the treatment of cancer, potentially improving clinical outcomes.

The review provides a comprehensive perspective on the anti-tumor potential of natural products by describing the outcomes of the main in vitro and in vivo experiments that have demonstrated some effects on various forms of cancer. Furthermore, this study summarizes the six directions of their anticancer activity by summarizing the mechanisms by which these agents affect cellular proliferation, differentiation, apoptosis, angiogenesis, and metastasis. In particular, it includes (I) compounds that possess an innate antitumor effect; (II) reverse chemoresistance; (III) inhibit metastasis; (IV) act as cancer immunotherapeutic; and (VI) the development of novel drug delivery systems for natural products. These natural products were chosen due to their potential anticancer properties as well as their substantial evaluation of chemotherapeutic effectiveness. It will provide the reader with an updated perspective on natural therapies and their potential application in cancer treatment in the future. As a result, this article offers a broader range of options for researchers interested in developing new alternatives for treating and preventing cancer, a topic that continues to grow in importance throughout the world.

## 2. Natural Products (NPs) against Cancer

Natural medicine refers to substances that are produced naturally by living organisms, such as plants, insects, animals, aquatic organisms, and microbes, and possess pharmacological or biological properties [19]. Natural products are precious gifts from nature that can be used for the prevention and treatment of illnesses in humans. Therefore, they are of vital importance and play an irreplaceable role in the development and design of drugs. Since ancient times, natural products have been used to treat human diseases. Recent research indicates that natural products still have the potential to be applied in drug development. The latest review by David J. Newman and Gordon M. Cragg revealed that 32% of all small-molecule drugs approved between January 1981 and September 2019 were natural products and their derivatives [20,21]. Moreover, from 1981 to 2014, 51% of all the 1211 new small molecule drugs approved worldwide were compounds derived from natural products [21]. In addition, a report by Eric Patridge and colleagues, published in 2016, revealed that the US Food and Drug Administration (FDA) approved 547 natural products and their derivatives for use as medications across the years (1827 to 2013) [22]. They are used to treat a number of diseases, primarily cancer, bacterial infections, and hypertension. The same study found that 68% of all 136 small-molecule anticancer drugs available from 1940 to 2014 were natural products based.

One of the most important sources of biologically active compounds is the plant kingdom. Currently, there are more than 350,000 vascular plant species registered in the world, and new species are being added every year [1]. It remains a vast and unexplored field of study that offers many opportunities for drug discovery. Plants can be used therapeutically in a variety of forms, such as tea, extracts, and dyes. In addition, their active compounds may be isolated and used as medicines or as precursors for synthetic and semi-synthetic drugs. There is a large list of phytochemicals (i.e., chemical compounds produced by plants) with therapeutic activity, including terpenes, alkaloids, essential oils, flavonoids, gums, and a variety of primary and secondary metabolic compounds [23]. In the past few decades, phytochemicals have been extensively studied for their potential anticancer properties, with the objective of using them in cancer treatment modalities such as chemotherapy and targeted therapy. For example, berberine is a secondary metabolite produced by plants, commonly found in the roots, rhizomes, and stem barks of Chinese herbs and plants of the *Berberis* genus. Numerous preclinical and limited human studies have demonstrated that berberine exerts beneficial biological activities against a variety of human diseases, including inflammation, metabolic dysfunction, depression, cardiovascular diseases, neurodegenerative diseases, and different types of cancers [24,25].

Since natural products exhibit unique properties as compared to conventional synthetic molecules, they are both advantageous and challenging in drug discovery. NPs are characterized by a wide range of scaffolds and a high degree of structural complexity [26]. These molecules generally have a higher molecular mass, a higher number of sp3 carbon and oxygen atoms, fewer nitrogen and halogen atoms, higher numbers of H-bond acceptors and donors, a lower calculated octanol–water partition coefficient (cLogP values, which indicate a higher degree of hydrophilicity), and a significantly higher molecular rigidity than synthetic compounds. A number of these differences can be advantageous; for example, the higher rigidity of NPs may be helpful when tackling protein–protein interactions in the drug discovery process. In fact, NPs are one of the most important sources of oral drugs beyond Lipinski’s rule of five [6]. Over the past 20 years, the increasing molecular mass of approved oral drugs illustrates the growing importance of drugs that do not comply with this rule. The structural optimization of NPs has been influenced by evolution to serve specific biological functions, such as regulating endogenous defense mechanisms and interacting (often in competition) with other organisms, which is why they are important for treating diseases such as cancer and infectious diseases. Additionally, the traditional use of these substances may provide insight into their efficacy as well as safety. Furthermore, the NPs pool contains a wide range of ‘bioactive’ compounds compared to typical synthetic small molecule libraries that cover a narrower chemical space [27].

Natural products account for approximately 50% of anti-tumor drugs. They may be derived from plants or can be semi-synthetic chemicals. Currently, many plant-based antitumor drugs are in clinical use, such as taxanes (e.g., Taxol), vinblastine, vincristine, and podophyllotoxin analogs. Robert Noble and Charles Beer discovered vincristine and vinblastine vinca alkaloids in the 1950s from the leaves of *Catharanthus roseus* (Madagascar periwinkle) [28]. Furthermore, the Taxus genus produces taxane-derived drugs. In 1971, paclitaxel, which is known commercially as Taxol^®^, was first isolated from *Taxus brevifolia* Nutt (Pacific yew). This is one of several diterpene taxanes that are used as the main chemotherapeutic agent against several forms of cancer, including ovarian, lung, and breast cancers. During the 1980s, another semi-synthetic taxane drug called docetaxel (Taxotere^®^) was isolated from *Taxus baccata* (European yew). Recent developments in chemical modification have focused on podophyllotoxin analogs, particularly teniposide and etoposide, two semi-synthetic antitumor agents. In 1880, Podwyssotzki discovered podophyllotoxin in the North American mayapple *Podophyllum peltatum* L. Furthermore, podophyllotoxin has also been found in *Podophyllum emodi* (Indian podophyllum) [29]. In 1966, etoposide was synthesized for the first time and was approved by the United States Food and Drug Administration (FDA) in 1983 as an anticancer agent [30].

**Figure 1:**
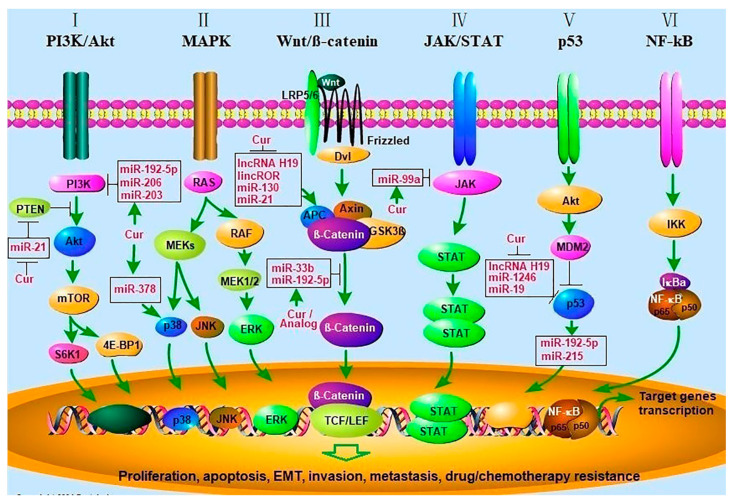
Curcumin inhibits cancer progression by regulating many signal pathways. (I) Akt/PI3K/mTOR signaling pathway. PTEN inhibits Akt activation by PI3K, mTOR phosphorylates p70S6K1 (S6K1), and 4E-BP1 which activate cell growth and survival pathways. Curcumin inhibits the Akt/PI3K/mTOR pathway by increasing PTEN expression through decreased miR-21 levels, and by inhibiting PI3K activity by upregulating miR-192-5p, miR-206, and miR-203; (II) MAPK signaling pathway. Signal cascades lead to activation of the MEKs, which subsequently activate the ERK1/2, p38, and JNK cascades and initiate the transcription of genes. Curcumin activates the p38 MAPK pathway by upregulating miR-378, which increases p21/27, cleaves caspase-3,9, and decreases Bcl-2 and MMP2/9. (III) Wnt/β-catenin pathway. The wnt molecule binds to both Frizzled and LRP5/6 receptors to cause the Axin/APC/GSK3β complex to dissociate. As a result, β-catenin is phosphorylated and translocated to the nucleus where it binds TCF/LEF co-transcription factors, which stimulate the transcription of Wnt-response genes. Curcumin inhibits Wnt/ β -catenin signaling through inhibition of lncRNA H19, lincROR, miR-130, 21, and upregulation of miR-192-5p and miR-33b; and (IV) JAK/STAT signaling. This pathway is activated when a ligand binds to a receptor, which communicates signals downstream STATs, whereas STATs are transcription factors that regulate gene expression; (V) p53 signaling pathway. The activation of MDM2 by AKT may inhibit the antitumor activity of p53. Curcumin inhibits lncRNA H19, miR-1246, and miR-19 to increase p53’s anti-tumor activity; (VI) NF-kB signaling pathway. This signaling cascade results in the phosphorylation of IkBα, which is then degraded by the proteasome. This allows the NF-kB/p65/p50 complex to translocate to the nucleus in order to facilitate transcription. This figure was reproduced from [32] (licensed under creative commons license).

Natural products can be used for chemoprevention and chemotherapy since it effectively suppresses cell proliferation, regulates the cell cycle, and interferes with several tumorigenic signaling pathways, such as phosphoinositide 3-kinase (PI3K), matrix metalloproteinase (MMP), MAPK/ERK known as the Ras-Raf-MEK-ERK pathway, toll-like receptor (TLR) pathway, and AKT pathway. Additionally, natural products may stimulate DNA repairing mechanisms through the action of p21, p27, p51, and/or p53 gene products, such as Bax, Bak, and Bid proteins, which cause the synthesis of protective enzymes such as caspases 3, 7, 8, 9, 10, 12, and modulate antioxidant enzymes such as GST, GSH, and GPx. The chemo-preventive effects occur by inhibiting important events involved in tumor initiation such as ROS removal, enhancing DNA repair, and eradicating transformed cells through immunosurveillance. Furthermore, these effects are exerted through inhibition of proliferation and immunotargeting of altered cells, as well as hindering tumor progression by promoting differentiation, inhibiting angiogenesis, and inducing apoptosis [31]. Curcumin has been one of the most studied phytochemical and its anticancer mechanism is shown in Figure 1.

### 2.1. Natural Products against Chemoresistance

**Figure 2:**
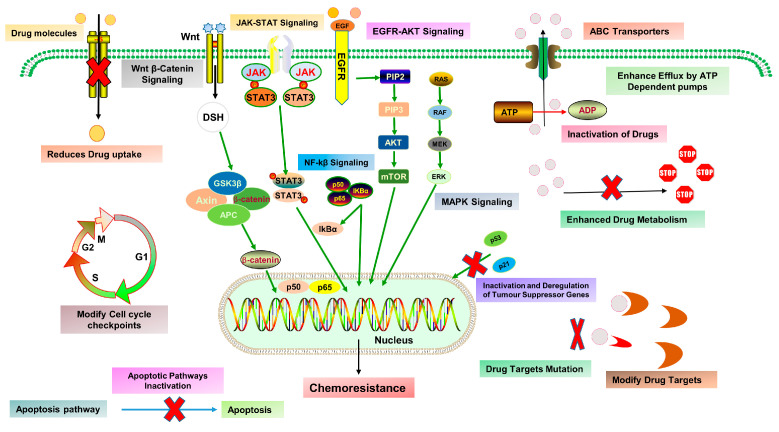
The mechanism of chemoresistance in cancer cells may include a variety of molecular mechanisms, such as regulating drug influx and efflux through ABC transporters, inhibiting cell death, altering drug targets, regulating epigenetic factors, inactivating chemotherapeutic agents, inactivating tumor suppressor genes, modifying DNA repair processes, and modulating growth factor signaling (adapted from [33], License Number: 5433490420433, License date: 21 November 2022).

Cancer develops from the abnormality of different molecular paths; therefore, treatment with current standard chemotherapeutic agents still remains less effective due to targeting of one or a few pathways. As a result, higher doses of conventional drugs are required to eliminate the tumor, which evokes harmful effects even worse than the condition itself. Although these agents are understood to kill tumor cells, they also trigger many survival pathways, which eventually induce cancer drug resistance, generally termed chemoresistance, which develops when cancer cells establish the resistance capability against chemotherapy and are categorized into two types: intrinsic (preexistent) and acquired (drugs induced). Chemoresistance development in tumor cells occurs via different molecular paths, e.g., regulation of drug influx and efflux via ATP binding cassette (ABC) transporter, epigenetic factors, hindering cell death, altering metabolism and degradation of drugs, and deactivation of chemotherapeutic drugs, mutations of drug targets, improved DNA repair and modifying growth factors signaling. These factors may act alone or in combination with different signaling pathways (Figure 2) [33].

Over the decades, the Food and Drug Administration (FDA) has approved about 300 chemotherapeutic drugs for the treatment of cancer, including taxol, vinca and its derivatives, Platinium analogues, 5-fluorouracil, bevacizumab, erlotinib, nivolumab, ipilimumab, sunitinib, and olaparib, with other different chemotherapeutic drug combinations. However, most of them are highly toxic and induce resistance, which ultimately results in tumor recurrence and metastasis, limiting their anticancer activities due to the development of adaptive resistance by targeted tumor cells and non-specific toxicities towards normal cells [34]. Therefore, better alternatives which are safer and more effective are required to overcome chemoresistance and subsequently sensitize tumor cells to chemotherapeutic agents. Chemosensitization is a widely used approach in which a drug’s activity is augmented using other drugs to suppress chemoresistance. However, the chemosensitizing agent needs to be safe and possess multitargeted inhibition potential of various chemoresistance pathways. The combinatorial chemotherapy produces synergistic effects by suppressing proteins, genes, and pathways involved in chemoresistance and concurrently modulates molecular targets of cancer to improve conventional drug therapy. Intriguingly, in vitro, ex vivo, in vivo, and clinical results suggest that phytochemicals are less toxic, safer, easily available, and highly effective with multi-targeting potential against different malignancies and also reduce the toxicity of conventional chemotherapy by increasing sensitivity of tumor cells [35].

Lupeol has been shown to sensitize cancer therapy-resistant cells by modulating various inflammatory cytokines such as IL-6, TNF-α, IFN-γ and also BAX, BCL-2, FAS, Caspases, Survivin, and PI3K-AKT-mTOR pathways and Cyclins, CDKs, P21, P53, and PCNA molecules, which are involved in cell cycle regulation [36]. Shikonin, a natural naphthoquinone, induces necroptosis in various cancer cell lines such as glioma, osteosarcoma, and hematopoietic cells by enhancing RIP1 and RIP3 and mROS, and also effectively overcomes P-gp, Bcl-2, Bcl-xl, MRP1, and BCRP1 drug resistance [37]. Similarly, Pterostilbene induces apoptosis, autophagy, S-phase cell cycle arrest, and inhibiting MDR1 expression via downregulation of RAGE/P13k/Akt pathways [38]. Epigallocatechin gallate induces G2/M cell cycle arrest and also suppresses MDR levels [39]. Plumbagin inhibits multiple molecular pathways such as cell cycle arrest, antiangiogenic, apoptotic, and autophagic pathways; regulates associated genes such as Akt, STAT3, and Nf-kβ; induces ROS; suppresses glutathione; and causes breakage of DNA strands [40]. Quercetin, a natural flavonoid, has been shown to facilitate cell death and chemosensitivity in pancreatic cells by RAGE/P13/AKT/mTOR axis [41]. Muthusamy et al. showed that ferulic acid reverses the P-gp mediated MDR by inhibiting P13K/AKT/Nf-kβ pathway in a tumor xenograft model [42].

### 2.2. Natural Products against Metastasis

Metastasis is a highly complicated process that involves primary tumor mass, circulating tumor cells (CTCs), and abnormal tumor microenvironments and is a leading contributor to cancer-linked deaths. The spread of metastasis to a specific organ is determined by various factors: for example, observing patterns of circulation, such as receiving direct blood from the primary site, e.g., hepatic metastasis happens in patients with colorectal cancer. Vascular permeability promotes extravasation at tumor sites, which further increases the probability of metastasis because instead of being autonomous, tumor cells become involved in bidirectional communication with metastatic microenvironments and modify the antitumoral immunity, genomic stability, extracellular milieu, survival signaling, chemoresistance, and proliferation activities. Despite improvements in advanced technologies, metastasis remains a major problem to resolve. The main therapeutic objectives are to prevent metastasis in high-risk patients, reducing advanced lesions, and avoiding additional metastasis with the limited disease [43].

Unlike conventional anticancer therapeutics, phytochemicals modulate multiple pathways during tumor initiation, progression, and metastasis development. Phytochemicals modulate primary epigenetic mechanisms regulating genes in metastasis such as DNA methylation, modification of histones, and/or non-coding RNA (ncRNA) associated multigene silencing. Regarding the complexity of metastatic tumors, multiple drugs can be used, such as the therapeutic approach used against (HIV) virus, because monotherapy can produce some initial responses. However, it lacks durability, so effective and durable therapy is required which minimizes the formation of resistance and improves durability. Phytochemicals can provide a better option due to their compatibility with multiple agents, which needs to be explored in detail. Some of the commonly explored and proven phytochemicals with anticancer and antimetastatic activities are apigenin, allicin, α-carotene, baicalein, and berberine curcumin, wogonin, formononetin, gambojic/ursolic/ellagic acid, papaya pectins, sulforaphane, and isothiocyanates [44].

**Figure 3:**
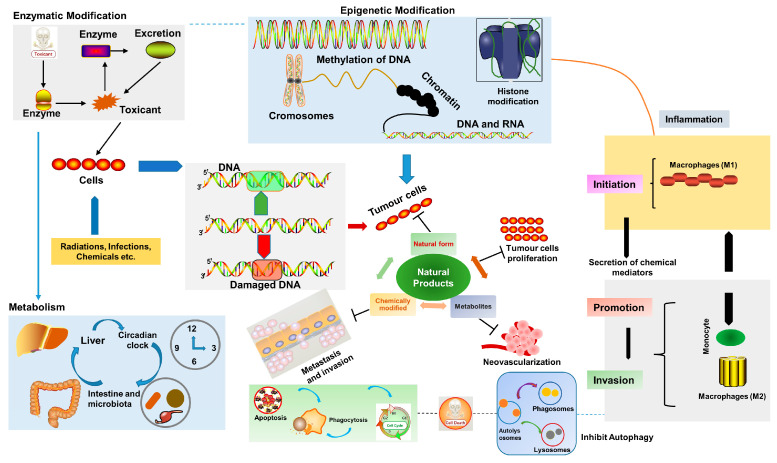
Several aspects of the development of cancer and the potential role of natural products in cancer prevention and therapy (adapted from [46], licensed under creative common attribution license).

In the recent past, increasing proof of toxicity related to chemotherapeutic agents, even at the smallest therapeutic doses, has paved the way for exploring a nature-based approach and various medicinal plant extracts/phytochemicals have been utilized at different stages of cancer progression [45]; their general mechanisms are shown in Figure 3.

### 2.3. Natural Products and Cancer Immunotherapy

Tumor immunotherapy is relatively safe and effective, such as immune checkpoint blockade, and is considered a major breakthrough in recent decades. Both types (innate and adaptive) of anti-tumor immune responses can be initiated. Tumor-suppressing natural agents exert their action by upregulating immune responses in the tumor microenvironment, primarily via M1/M2-type macrophages. Recently, Sun et al. found that resveratrol can inhibit the polarization of human monocyte-derived macrophages into M2, and they also observed that lung cancer medium induced polarized M2 macrophages were modified to M1 polarized macrophages after resveratrol treatment by increasing IL-12 and TNF-ɑ and reducing IL-10 levels. Additionally, M2 macrophage markers’ expression was decreased, such as MRC1, chiI3, CCL24, and Retnla [47]. Moreover, Loo et al. successfully increased the immunity in postoperative patients with Rhodiola algida mixture by increasing the expression of IL-2, IL-4, GM-CSF, and mRNA content, thereby decreasing the increased risk of oral cancer [48].

Improving the functional activity of dendritic cells in cancer patients can significantly improve the treatment outcome by activating T cells, as mature dendritic cells secrete IL-12, which acts on T cells and encourage Th1 cell differentiation [49]. Chang et al. showed that the treatment of DCs with Astragalus and Codonopsis polysaccharide could promote the proliferation of CD4+ and CD8+ T cells in mice, and DC-based vaccine increased the anti-metastatic efficacy in 4T1 cells bearing mice by increasing the expression of CD40, CD80, and CD86 levels on the surface of DCs [50]. Similarly, it has been shown that shikonin, combined with B16 tumor cell lysate, could activate DCs to a more mature state with increased surface levels of CD86 and MHC II, which activates Th1 cells and improve its functions. Subsequently, the shikonin-tumor cell load DCs vaccine improved the cytotoxicity of splenocytes to cancer cells, hampered tumor growth, and increased mice’s survival [51].

NK cells help in differentiating stem cells and undifferentiated cancer cells by secreting IFN-γ and TNF-α, which limit the tumor growth by remodeling/regulating the tumor microenvironment. Natural products activate NK cells to stop tumor growth and metastasis [52]. Wu et al. have shown that lupeol can activate NK cells, which further activates the PI3K/Akt and Wnt/β- catenin pathway by increasing the expression of IFN-γ, PFP, and CD107a, as a result, inhibiting the proliferation of gastric cells (N87, HGC27 and BGC823) [53]. In addition, Hou et al. reported that total flavonoids obtained from *Hippophae rhamnoides* increased the cytotoxicity of NK cells against tumor cells by increasing the expression of NKp44, NKp46, granzymes B, and perforin [54].

MDSCs, a heterogeneous group of cells, promotes tumor progression, angiogenesis, and metastasis to healthy cells/tissues. Recently, Zhou et al. studied the effects of icariin on 4T1-Neu xenograft mice and noted that the number of MDSCs decreased in the spleen of the mice. Furthermore, icariin also restored the secretion IFN-γ by CD8+ T cells in the mice and reduced nitric oxide and ROS levels in MSDCs in vivo, while in vitro treatment differentiated into dendritic cells and macrophages [55]. The Shugan Jianpi formula has recently been reported to effectively regulate tumor microenvironment by suppressing CD8+ cells apoptosis, reducing MDSC proliferation and tumor activity in 4T1 tumor cells bearing mice [56].

Peripheral T lymphocytes CD4+ and CD8+ play a key part in anti-tumor immunity, and CD4+ T cells help in recruiting CD8+ T cells, while CD8+ T cells directly attack and kill tumor cells. CD4+ T cells can be differentiated into many subgroups of cells, such as Th1, Th2, Th9, Th17, Tfh, Treg cells, etc. The Th1/Th2 imbalance plays a vital role in the occurrence of tumors [57], and natural products increase Th1 development, which leads to the suppression of tumors. Wei et al. reported that with the addition of Tetramethylpyrazine phosphate (plant extracts) to the culture of lung cancer patient-derived PBMC cells and healthy subject cells, the expression of IFN-γ and IL-2 was increased, and Th2 cytokines decreased in patient-derived cells; the killing efficiency of macrophages was also improved [58]. Eckol is derived from *Ecklonia cava*, brown algae, and has shown potent antiproliferative and immunomodulatory activities. In a recent in vivo antitumoral study, the cytotoxic effects of eckol were evaluated by using sarcoma S180 xenograft model. The results stated that all the pro-apoptotic and antiproliferative proteins were upregulated, eckol also stimulated (MPS) and activated dendritic cells, which produced Th1 responses and enhanced CD4+/CD8+ T lymphocyte ratio, as a result cytotoxic T lymphocytes responses were increased [59]. Takei et al. observed that Ginsenoside promotes the transformation of naive T cells into Th1 cells by acting on DCs, increasing IFN-γ level, and in CTL assays, it was found that the Ginsenoside induced more IFN-γ than the mature DCs, which indicates that combined treatment may exert strong immunogenic response [60].

In recent times, cancer immunotherapy has gained some success by engineering antigen-specific T cells, e.g., TCR-T and CAR-T cells. Especially in hematological cancer, the CAR-T targets the CD19 and has been used and allowed for clinical use by the US FDA. Similarly, TCR-T cells have been used to treat cancer; however, MHC-restriction hinders their potential clinical use. There are no reports available which show the use of CAR-T cells and TCR-T cells combined with natural products, but this is an emerging area of research and needs to be explored, and we might obtain some successful outcomes in treating various cancers [61].

Astragalus polysaccharides have been shown to inhibit the proliferation of Treg cells in a dose- and time-dependent manner by reestablishing the cytokine balance and decreasing the expression of Foxp3 in the microenvironment of HCC. Moreover, Astragalus polysaccharides can block the (SDF-1) or its receptor (responsible for recruiting Treg cells into the HCC microenvironment) via the CXCR4/CXCL12 signaling pathway, thus inhibiting Treg cells migration [62]. Astragalus polysaccharides also act as an immunomodulatory agent by activating the TLR4 pathway, which inhibits TGF-β, resulting in a decrease in the number of Treg cells [63]. Similarly, Radix glycyrrhizae polysaccharides have been shown to downregulate the number of Treg cells in the microenvironment of H22 tumor xenograft mice by decreasing the expression of Foxp3 in Treg cells while upregulating the Th1/Th2 ratio in serum; as a result, inhibiting tumor growth [64].

Recently, targeting immune checkpoint molecules (PD-1 and CTLA4) has been considered a promising approach to treat various diseases such as cancer. These checkpoint molecules play an essential role in regulating immune homeostasis [65]. Currently, immune checkpoint molecules include programmed death receptor 1 (PD-1), cytotoxic T-lymphocyte associated antigen 4 (CTLA4), lymphocyte activation gene 3 (LAG3), and T cell immunoglobulin mucin 3 (Tim3). Presently, very little work is done on using natural products to target immune checkpoint molecules, and this research area needs more exploration [66]. Zhang et al. showed that Qiyusanlong decoction suppressed tumor growth in Lewis lung carcinoma-bearing mice by decreasing both mRNA and PD-1/PDL-1 protein levels in the tumor [67]. Similarly, another study revealed that Gegen-qinlian decoction increased the effects of PD-1 blockade in colon cancer via remodeling of intestinal microbiota, which demonstrated that phytochemicals could block PD-1 and thereby could be a possible approach for treating various cancers [68]. Zhao et al. showed that curcumin suppresses Treg cells (CD4+ CD25+) function by decreasing CTLA4 and Foxp3 [69].

### 2.4. Natural Products in Combination with Other Chemotherapeutic Drugs

Currently, combinatorial therapy has gained importance due to the administration of multiple chemotherapeutics with different biochemical targets, which increases efficacy as well as safety, and this concept is widely applied to different tumors. Many studies have reported the combination of widely used phytochemicals like (Resveratrol, curcumin, and thymoquinone) with other antitumoral drugs and have achieved significant success in preclinical studies [70]. Combination therapy reduces the toxicities associated with traditionally used chemotherapeutics such as doxorubicin (cardiomyopathy) and cisplatin (nephrotoxicity and immunosuppression), etc. The other advantage of combination therapy is overcoming drug resistance, which reduces adverse events by using low doses with the same efficacy and sometimes better pharmacological effects (synergism). Many preclinical studies and clinical trials are conducted to use phytochemicals as adjuvant therapy in various cancers. Chemoresistance has been commonly endured by many traditional chemotherapeutics, which is the main concern and hurdle in tumor therapy along with radiation resistance. Natural products are ideal candidates for chemosensitizing tumor cells and increasing the efficacy of existing drugs [71]. Silybinin has been shown to help doxorubicin in overcoming drug resistance in colorectal cancer by inhibiting GLUT1 expression. Many natural agents act as antioxidants, such as ROS scavenging/antioxidation, thereby reducing the toxicities related to the generation of free radicals. However, drug–drug interaction could be the main disadvantage of combinatorial therapy [72].

Curcumin has been used in combination with many chemotherapeutic drugs for the treatment of various cancers. For example, curcumin sensitizes tumor cells to 5-fluorouracil, which inhibits the tumor growth in HCT116 cells/xenograft model via inhibition of AMPK/UKL1 modulation of the AKT pathway [73]. Similarly, curcumin augmented doxorubicin’s anti-tumor activity by suppressing cell migration and inducing apoptosis in neuroblastoma SH-SY5Y cells via upregulation of p21 p53, and TIMP1 and downregulation of MMP2 [74]. Resveratrol and paclitaxel combination is reported to produce synergistic effects through the stimulation of TRPM2 channel in glioblastoma DBTRG cell line, which resulted in oxidative stress and induced apoptosis, which shows that resveratrol can be used in combination with other chemotherapeutic agents which can enhance the treatment efficiency [75]. Shen et al. reported that noscapine increases the sensitivity of cisplatin by modulating the cell cycle (G2/M phase arrest) and inducing apoptosis (downregulating XIAP and NF-kβ, while upregulating caspase-3) in cisplatin-resistant ovarian SKOV3 cells and xenograft in mice [76]. In addition, Neferine has been shown to increase the antitumor activity of cisplatin in lung cancer A549 cells via G1 phase cell cycle arrest, hypergeneration of ROS, upregulating Bax, BAk, p53, and c-myc levels while downregulating Bcl-2, FAK, VEGF, MMP-2/-9, and loss of membrane potential (ΔΨM) [77]. Wang et al. used cryptotanshinone in combination with paclitaxel against tongue squamous cell carcinoma (CAL 27 and SCC) cell lines, which increased the antitumor activity by inhibiting the migration and proliferation of cells as well as inducing apoptosis due to inhibition of JAK/STAT3 signaling pathway [78].

Similarly, Zhang et al. showed that a combination of formononetin and temozolomide could produce synergistic effects in C6 glioma cells through upregulation of Bax and caspase-3/-9 expression while downregulating Bcl-2 and MMP-2/-9 expressions [79]. Furthermore, Tseng et al. reported that aloe emodin enhanced the cytotoxicity of tamoxifen in different breast cancer cells (MDA-MB 231, MCF-7, HCC1954, and BT-474 cells) via suppression of Ras/ERK and PI3K/mTOR signaling pathway [80]. Similarly, a combination of capsaicin and docetaxel has been shown to have higher cytotoxicity against prostate cancer (LNCaP and PC3) cell lines and tumor xenograft in mice by inhibiting the growth and proliferation via reducing Akt, mTOR, and S6 phosphorylation while increasing (AMPK) phosphorylation [81].

Recently, Saikia et al. reported that combined use of heteronemin with cytarabine increases its sensitivity against leukemia (HL-60) cells by downregulating downstream targets of Ras signaling pathway such as AP-1, NF-kβ, AMPK, and c-myc; as a result it produces synergistic effects, inhibits the growth, and induces apoptosis [82]. Furthermore, the combined use of celastrol and triptolide has been shown to produce synergistic effects against various cancer models in vitro (H1299, H460, SKOV3, OVCAR3, Hela, SIHA, and SW480 cell lines) and in vivo studies (xenograft in mice) by inhibiting growth and proliferation through increasing ROS, G2/M phase arrest, decreasing Akt, surviving, and EGFR expressions [83]. Moreover, the combined use of berberine and sorafenib is effective in liver carcinoma because berberine increases the sensitivity of HCC (HepG2 and SMMC-7721) cells to sorafenib, which ultimately inhibits the growth and proliferation and induces apoptosis by increasing the expression of cleaved PARP and cleaved caspase-3, while decreasing the expression of Bcl-2 and VEGF markers [84]. Desai et al. studied the combined effects of biochanin A and Temozolomide against U-87 and T-98 MG cells and observed that the growth and proliferation of the cells were synergistically inhibited and the expression of p-p53 was increased, while the expression of p-Akt, EGFR, p-ERK, and MT-MMP1 was decreased [85]. Mangiferin increased the sensitivity of oxaliplatin towards MCF-7, Hela, and HT29 cell lines by increasing the expression of caspase-3, S-phase cycle arrest, and reducing Nf-kβ activation, which induced apoptosis [86].

In a recent study, dietary phytochemical fisetin was shown to produce synergistic effects in combination with sorafenib against cervical cancer (Hela) cells by activating DR5-mediated caspase-3/8 activity and mitochondrial apoptotic pathway [87]. In another study, the combined use of ursolic acid and temozolomide has been shown to produce synergistic effects in in vitro and in vivo studies in mice by overcoming temozolomide-resistance due to the downregulation of O6-methylguanine DNA-methyltransferase (MGMT), which resulted in an increase in cytotoxicity and inhibited the proliferation of the tumor cells, which indicates the potential of ursolic acid as a monotherapeutic anticancer agent as well as a chemosensitizer [88]. Similarly, a combination of lipoic acid and other antineoplastic drugs (5-FU and Doxorubicin) resulted in a decrease in p53-mediated stabilization of p21, which resulted in the synergistic killing of colorectal cancer cells [89]. Recently, Huang et al. showed that vitamin D sensitizes oral squamous carcinoma cells to cisplatin by inhibiting lipocalin-2 (LCN2) modulated Nf-kβ signaling pathway via ribosomal protein S3 (RPS3), which partially reversed the cisplatin resistance [90]. Moreover, the combined use of embelin (XIAP inhibitor) and celastrol (NF-kβ inhibitor) in acute myeloid leukemia (HL-60) cell line has been shown to produce synergistic and additive effects by downregulating COX-2 and survivin, which shows that simultaneous inhibition of the two tumor signaling pathways can improve the effectiveness of the chemotherapy [91].

The combination of withaferin A and oxaliplatin produced synergistic effects in pancreatic cancer (in vitro and in vivo studies) by increasing the generation of ROS, which ultimately led to the inhibition of the PI3K/AKT signaling pathway [92]. Yang et al. reported that tectorigenin increases the sensitivity of paclitaxel in paclitaxel-resistant ovarian tumor (SKVO3, MPSC1, and A27800) cell lines by activating caspase-3/-8/-9 and downregulating XIAP, Bcl-2, COX-2, FLIP, IkB, IKK, and Akt, which suggests that it increases the sensitivity and cytotoxicity by inactivating Akt/IkB/IKK/NF-kβ pathway [93]. Moreover, glabridin, a flavonoid, has been shown to reverse the P-gp mediated resistance to the conventional chemotherapeutic drugs (Doxorubicin and paclitaxel) in P-gp overexpressing breast cancer (MDA-MB-231/MDR1 and MCF-7/ADR) cell lines by suppressing P-gp and downregulating the functional activity of P-gp- ATPase and thereby reversing the multidrug resistance [94]. Similarly, Xia et al. showed that gambojic acid promotes gemcitabine’s sensitivity in pancreatic cancer (in vitro and in vivo studies) by inhibiting the ERK/E2F1/RRM2 signaling pathway, which decreased the proliferation and growth of tumors. Moreover, the study also indicated that gambojic acid can be used as a monotherapeutic agent, as well as a chemosensitizer [95]. In addition, the combined use of forbesione with 5-fluorouracil produced synergistic effects in inhibiting growth and proliferation in cholangiocarcinoma cancer (in vitro and in vivo studies using Ham-1 cells), through downregulating Bcl-2 and increasing expression of p53, Bax, Apaf-1, and caspase 3/-9, which induces apoptosis and results in the killing of the tumor cells [96]. Similarly, Su et al. showed that osthole enhances cisplatin’s effects by suppressing NRF2 expression and blocking the PI3K/Akt signaling pathway, thus inhibiting the progression of cisplatin-resistant cervical cancer (SiHa/cDDP) cells in vitro and in an in vivo xenograft model [97].

## 3. Natural Products against Some Common Types of Cancer

### 3.1. Lung Cancer

Lung cancer is highly occurring and a standout amongst the most widely recognized human malignancies in both developed and developing nations, with 2.1 million new lung cancer cases and 1.8 million deaths anticipated in 2018, nearly one of every five (18.4%) malignancy deaths [98,99]. Even though various treatment alternatives are available, including surgery, radiotherapy, chemotherapy, and targeted therapy, half of all newly diagnosed cancers are now at an advanced stage, where the effects of treatment are constrained. Treatment in such cases might be restricted to palliative care, leading to a low 5-year survival rate. Lung cancer comprises a couple of subtypes; for example, adenoma-carcinoma (AdCa), the most widely recognized subtype in non-smokers and females; squamous cell carcinoma (SqCC), and small cell lung cancer (SCLC). A few instances of current treatment for malignant lung growth incorporate medical procedures, chemotherapy, and radiotherapy, whereby the choice of treatment relies upon the subtype and stage of lung cancer. Nevertheless, there is a dire need to find other alternative chemotherapeutic agents, either fabricated as a more effective drug or in combination with the current therapeutic agents to have a more prominent and synergistic anticancer activity. Recently, Aucubin has been reported to produce antitumoral effects in lung cancer (A549) cells by inducing cell cycle arrest at G0/G1 phase, inducing p53 activation, and also increasing the activity of the Fas/Fas-ligand system [100]. Similarly, Xu et al. reported that cytisine, a natural alkaloid, produces significant cytotoxic effects against lung cancer A549, NCI-H23, and NCI-H460 cells and in vivo (rat model) by inducing apoptosis via an increase in ROS and loss of membrane potential; increasing BAD, cleaving PARP, and cleaving caspase-3 expressions; and decreasing Bcl-2, pro-PARP, and pro-caspase-3. Moreover, phosphorylation of p38, JNK, and I-kB was increased, while there was a significant decrease in the phosphorylation of ERK, NF-kβ, and STAT3. Furthermore, cytisine arrested the cell cycle at the G2/M phase, which was related to the inhibition of the Akt signaling pathway [101].

In another study, Han et al. showed that L-securinine from dried leaves of *Securinega suffruticosa* inhibited the proliferation of A549 cancer cells, decreased the expression of DKK1 genes, and promoted the methylation of DKK1 promoter in comparison to 5-azacytidine [102]. Similarly, gedunin isolated from *Azadirachta indica* induced apoptosis and inhibited the growth and proliferation of lung cancer A549 cells by generating ROS, decreasing the membrane potential, and causing DNA damage via downregulation of PIK3CA, EGFR, AKT, and autophagy. Furthermore, gedunin also disrupted the interaction between Hsp90: Bcl-1:Bcl-2 [103]. In another study, vanillin, the main ingredient of *Vanilla planifolia*, suppressed the CSC-like behavior of lung cancer NCI-H460 cells by inducing Akt-proteasomal degradation and decreasing downstream CSC transcription factors (Oct4 and Nanog) [104]. Additionally, cepharanthine isolated from *Stephania cepharantha* inhibited the growth and proliferation of lung cancer H1299 and A549 cells by increasing ROS, loss of membrane potential, and cell cycle arrest [105].

### 3.2. Breast Cancer

In 2018, there were about 2.1 million newly diagnosed female breast malignant growth cases around the world, representing nearly one of every four tumor cases among women. Breast cancer is the most widely diagnosed cancer by far in most nations (154 of 185) and is also the primary source of cancer-related deaths in more than 100 nations. For malignant breast tumor treatment, numerous alternatives are provided, for example, medical procedures, hormonal treatment, radiation treatment, chemotherapy, and targeted treatment [106]. However, there are also certain limitations (i.e., narrow therapeutic index with non-specific toxic consequences for healthy tissues, increasing chance of infection, etc.), and also the severe side effects persist for months or years, even after treatment completion. Therefore, further studies are required to explicate the underlying fundamental mechanisms of breast cancer to develop new therapeutic strategies. Breast cancer is the collection of abnormal cells, presumably credited to the imbalanced proliferation of cells, apoptosis, and the cluttered autophagy regulation. Various natural products were accounted for as possible anti-cancer agents or thought of as immediate or indirect sources of new chemotherapeutic adjuvants to upgrade the efficacy or enhance the symptoms through autophagy regulation [107]. Higenamine has been demonstrated to increase the anticancer activity (apoptosis and G2/M cell cycle arrest) of cucurbitacin B in breast cancer (T47D and SkBr3) cells by inhibiting Akt and CDK2 [108].

In another study, Citral, isolated from *Cymbopogon citrates*, suppressed the growth and proliferation of 4T1 breast cancer cells implanted in the nude BALB/c mice [109]. Similarly, euphol inhibited the growth and proliferation of breast cancer (T47D) cells by inducing G1 phase cell cycle arrest via downregulation of cyclin D1 and upregulation of p21 and p27 expressions [110]. In another study, Reddy et al. demonstrated that strophanthidin produces dose-dependent cytotoxic effects against breast (MCF7), liver (HepG2), and lung (A549) cancer cells by attenuating MAPK, Wnt/β-catenin, and PI3K/Akt/mTOR signaling pathways [111]. Moreover, jatrophone isolated from *Jatropha isabelli* inhibited the proliferation and EMT of triple-negative breast cancer cells by interfering with the cell cycle and Wnt/β-catenin signaling pathways [112]. Deng et al. reported that rotenone induced apoptosis in MCF7 breast cancer cells by producing ROS, chromatin condensation, upregulating Bax, downregulating Bcl2, and cleaving PARP. Furthermore, cJNK and P38 MAPKs were activated, and ERK (1/2) signaling pathways were inactivated [113]. Similarly, Dhandayuthapani et al. demonstrated bromelain to be effective against breast cancer by promoting apoptosis via upregulation of c-JNK, p38 kinase, and activating caspase 3 and 9 [114]. In another study, flavokawain B induced apoptosis and inhibited the proliferation of breast cancer (MCF-7 and MDA-MB231) cells by upregulating various kinases such as p-Akt, p-JNK, p-CREB, p-p53, and p-WNK1 and downregulating Nf-kβ, COX2, MMP-9, GLUT1, and VEGFA [115].

### 3.3. Ovarian Cancer

Ovarian cancer is one of the leading causes of death associated with the female reproductive system in the Western world. Ovarian cancer is a standout amongst the most deadly gynecological diseases in the female reproductive system, influencing approximately one out of 75 women in the United States. Even though the first-line treatment may profit about 80% of patients with ovarian malignancy, 75% of those patients still experience tumor recurrence, which causes a major worry surrounding the treatment of ovarian cancer in patients. Cisplatin is the most generally utilized chemotherapeutic entity for treating ovarian cancer. Treatment dereliction and death in over 90% of patients with metastatic malady are believed to be brought about by drug resistance. Adverse reactions and resistance developed to platinum-based chemotherapy have turned into an obstacle for ovarian cancer treatment. Therefore, it is important to search for new compounds to treat cancer and reduce the associated side effects of the treatments.

Natural bioactive agents have received an increased consideration in cancer treatment lately [116]. For example, diarylheptanoid hirsutenone potentiates the TRAIL-induced apoptotic activity in ovarian cancer (OVCAR-3 and SKOV-3) cells by enhancing the activation of caspase-8 and BID dependent signaling pathways, which further activates caspase-3/-9, exhibiting the significance of hirsutenone in combination with TRAIL treatment [117]. Paclitaxel, first isolated from *Taxus brevifolia*, has been used in the management of lung, breast, ovarian, prostate, sarcoma, leukemia, and endometrial cancer; it acts by interfering in spindle function, stabilizing microtubules, inducing apoptosis, cell cycle arrest, and inhibiting the growth of tumor cells [118]. Moreover, barbamine isolated from Berberis amurensis effectively inhibited the growth of cancer in vitro (SKOV3 and Es2 cell lines) and in vivo (SKOV3 xenograft model) by inducing apoptosis and targeting the Wnt/β-catenin pathway [119]. Recently, Nobilietin has also been shown to produce strong antitumor effects in ovarian cancer (HOCCs) cells by increasing the expression of cleaved PARP in a dose-dependent manner and inhibiting proliferation, causing DNA damage and inducing apoptosis. Many in vitro and in vivo studies have demonstrated that various flavonoids produce potent cytotoxic activities by inducing apoptosis, cell cycle arrest, and inhibiting angiogenesis [120].

### 3.4. Colon Cancer

Colorectal cancer (CRC) is prominent amongst the most occurring tumors worldwide, and as of late, it is the third-highest contributor to cancer-associated deaths in the United States of America and China [121]. It represents over 90% of the malignant tumors of the large bowel, and the other 10% comprises lymphoma and squamous cell carcinoma. The occurrence of colorectal cancer is continually increasing due to weak prognosis in patients possessing widely metastasized tumors, and the fundamental underlying mechanism of metastasis is not completely clear [122]. A few epidemiological investigations have exhibited the relationship of colon tumor growth with dietary propensities, for example, low fiber diet, high fat intake, and low calcium/micronutrient consumption [123]. The inverse correlation between the utilization of vegetables and fruits with different cancers has led researchers to examine the advantages of dietary elements in chemoprevention [124]. Recently, Morin has been shown to induce apoptosis and inhibit the growth of colon cancer (SW480) cells through the generation of ROS and decrease in mitochondrial membrane potential. Furthermore, it increased PARP, Bax, and cleaved caspase-3/-8/-9 expressions [125]. In another study, inflexinol isolated from *Isodon excisus* has shown the potential to strongly inhibit the growth and proliferation of colon cancer cells by inducing apoptosis through inactivation of NF-kβ in in vitro (SW620 and HCT116 cell lines) and in vivo (SW620 xenograft) rat model [126]. In addition, strychnine from *Nux vomica* has shown significant antiproliferative effects in vitro (SW480, Lovo, and DLD1) and in vivo (DLD1 xenografted model) by targeting the Wnt/β-catenin pathway by enhancing APC and decreasing β-catenin and c-Myc. Furthermore, the expression of DKK1 was also enhanced, known for negatively regulating the Wnt/β-catenin pathway [127].

In another study, Gao et al. showed that berginin significantly suppressed the proliferation of HCT116 cells by accumulating intracellular ROS, DNA damage, and G1 phase arrest through inhibition of PI3K/AKT/mTOR pathway [128]. Similarly, psoralidin inhibited the viability and proliferation of SW480 cells by inhibiting the Nf-kβ and Bcl-2/Bax signaling pathway [129]. In a recent study, matrine was also shown to produce antitumor effects in vitro against multiple colorectal cancer (LS174T, SW1116, Caco-2 and RKO) cells and in vivo in (LS174T xenografted) mice via G1/G0 phase arrest, reducing Bcl-2 and caspase-3, while upregulating cleaved caspase-3 and Bax expressions [130]. Magnolol isolated from *Magnolia officinalis* shows significant antiproliferative effects in vitro and in vivo against HT-29 and CT-26 cell lines by inducing apoptosis through intrinsic and extrinsic pathways inhibiting PKCδ/NF-kβ signaling pathway [131]. Similarly, Mi et al. showed that imperatonin isolated from *Angelica dahurica* suppresses the tumor growth, proliferation, and angiogenesis of human colon cancer (HeLa, Hep3B, and HCT116) cells through HIF-1α targeting through mTOR/p70S6K/4E-BP1 and MAPK signaling pathways [132]. Moreover, aloperinole is an alkaloid extracted from the leaves of *Sophora alopecuroides* which produces potent cytotoxic effects against colon cancer HCT116 cells by inducing apoptosis and inhibiting the proliferation by G2/M phase cell cycle arrest and increasing the expression of Bax, p21, and p53, while decreasing Bcl-2, CD1, and B1. Furthermore, it also inhibited PI3K/Akt and JAK/Stat3 pathway [133]. Many preclinical and clinical studies have indicated that bioactive components of fruits and vegetables may help in the deterrence of colon cancer.

### 3.5. Brain Cancer

Brain tumors represent 85–90% of all significant CNS tumors. Glioblastoma (GBM) represents around half of all aggressive brain tumors and is related to low survival. Multimodal treatments, including surgery, pursued by adjuvant chemoradiation therapy (CRT) with temozolomide (TMZ), is a standardized treatment for GBM. The TMZ increases overall survival (OS) from 7.7 to 13.5 months and from 7.9 to 10.0 months in the GBM patients, which is extremely poor [134]. This poor survival is likely due to numerous variables, including systemic toxicity due to higher TMZ dosages, BBB impermeability, CRT resistance, and progression of refractory tumors [135]. Hence, identifying a novel chemotherapeutic agent that can modulate the BBB, restrain tumor development, and stop tumors recurrence is vital for better patient prognosis. In recent studies, ginkgetin extracted from Cephalotaxus fortune has been shown to effectively inhibit the growth, invasion, and proliferation of medulloblastoma (Daoy and D283 MB) cell lines by arresting cell cycle at (G2/M phase), and also inhibit Wnt/β catenin signaling pathway [136]. Similarly, Cao et al. reported that toosendanin shows higher cytotoxic effects in vitro and in vivo against glioblastoma multiforme (U87 and C6 cell lines) by inducing apoptosis via upregulation of ERβ and functional activation of p53 [137]. Recently, Noman et al. isolated prestegane B from *Thymelaea microphylla*, evaluated it for antiproliferative properties against C6 and Hela cell lines, and found that it shows higher antiproliferative and radical scavenging ability than some of the other known drugs [138]. Extracts of Viscum album are widely used as complementary medicine for cancer therapy, and recently some of the extracts such as aviscumine, iscador Qu, and ML-1 have been shown to regulate the expression of genes associated with the cell migration, invasion, adhesion, and even cell architecture formation in glioma cells by inhibiting TGF-β, SMAD2, and MMP-2/-9. These ingredients have the potential to be used as an adjuvant therapy for the treatment of glioblastoma [139].

### 3.6. Liver Cancer

Liver cancer was the sixth most commonly reported cancer and the fourth leading cause of cancer-related deaths worldwide in 2018, with around 841,000 new cases and 782,000 deaths every year. Liver cancer is the most widely recognized tumor of the digestive system, with a high mortality rate. Based on conformational evidence, numerous natural dietary compounds are potential hotspots for the prevention and management of liver malignancy [140]. Hepatocellular carcinoma (HCC) is the primary type of malignant liver tumors (70–80%), trailed by intrahepatic cholangiocarcinoma. The major risk factors for liver cancer are hepatitis B/hepatitis C disease, alcohol usage, aflatoxin B1, and metabolic issues. Liver cancer is mostly aggressive with a poor prognosis, having a low five-year survival rate below 9% [141]. Proper interventions such as liver resection, transplantation, and percutaneous removal are considered as the best approaches with curing potential for liver cancer. However, because of various injuries and extrahepatic metastasis, merely 20% of liver cancer patients are appropriate for a medical procedure. On the contrary, chemotherapeutic medications for liver cancer are constrained, and sorafenib is the most well-known medicine. The phase III clinical trials exhibited that sorafenib could increase the overall survival and progression time. However, its clinical gains are modest, and it was noted that sorafenib was only valuable for around 30% of patients, and resistance developed in half a year. Moreover, other issues such as hepatotoxicity, drug resistance, recurrence, and other unwanted side effects exist in present treatments, which compels researchers to look for an alternative therapy [142].

Recently, echinoside A and ds-echinoside, isolated from pearsonothuria graeffei, inhibited tumor growth and proliferation in the HepG2 cells by G0/G1 phase arrest and induced apoptosis via the mitochondrial pathway [143]. In another study, oleuropein has been shown to effectively inhibit the cell viability and proliferation of hepatocellular carcinoma (HepG2) cells by inducing apoptosis through ROS production, increasing Bax, and decreasing Bcl-2. Moreover, oleuropein also targeted the PI3k/Akt signaling pathway [144]. Similarly, crocin isolated from saffron has been shown to produce autophagic apoptosis and inhibit the growth and proliferation of human hepatic cancer (HCCLM3 and HepG2) cells via upregulation of LC3-II and constraining the functional activities of key proteins involved in Akt/mTOR signaling pathway such as p-Akt, p-mTOR, and p-p70S6K [145]. In addition, *Alpinia oxyphylla* extracts inhibited the proliferation and tumor growth in hepatocellular carcinoma (HepG2, Hep3B, Bel-7402, and SMMC-7721) cell lines and also in vivo Hep3B xenograft by increasing Bax and caspase-3/-9 expressions and decreasing Bcl-2 levels. Furthermore, it also upregulated PTEN and downregulated PI3k and inhibited the phosphorylation of Akt [146]. Alpidine, a cyclic depsipeptide isolated from *Aplidium albicans*, has produced antiproliferative effects in various tumors such as melanoma, breast and lung cancer via activation of JNK/p38 MAPK pathway and induces apoptosis [147]. Recently, corilagin has been shown to produce significant cytotoxic effects in vitro and in vivo against liver cancer SMMC7721 cells via G2/M phase cycle arrest, and by downregulating p-Akt and cyclin B1/cdc2 and upregulating p-p53 and p-p21^Cip1^ [148].

### 3.7. Head and Neck Cancer

Head and neck squamous cell carcinoma (HNSCCs) is among the sixth most common malignancies globally and accounts for 90% of all the head and neck tumors occurring at different anatomical sites. Alcohol consumption, tobacco use, and infection with the human papillomavirus (HPV) are thought to be the oncogenic drivers. Treatment options involve surgical resection, radiotherapy, and targeted chemotherapy, but are mostly ineffective and subsequent relapse frequently occurs due to tumor resistance. Along with treatment failure, these therapies result in higher morbidity and reduced quality of life due to their severely toxic nature [149]. This inadequate therapeutic response can be associated with alterations in intracellular signaling pathways and significant changes in the extracellular tumor microenvironment. Therefore, a better, safe, and effective alternative is needed to address these challenges to improve the treatment standard and reduce the treatment cost. Over the decade, various novel approaches have been considered to understand and target specific disease targets but have fallen short of achieving clinically significant results [150].

Phytochemicals have shown enormous potential in chemosensitizing and inhibiting the growth of HNSCCs in various in vitro and in vivo models. Recently, active constituents of *Piper methysticum* (kava) such as flavokawain A, flavokawain B, and yangonin have been shown to exhibit antiproliferative effects against OSCC cancer (BICR56 and H400) cells but are non-toxic to normal keratinocytes (OKF6), showing promise for in vivo translation. Additionally, Pycnogenol, isolated from pine bark, inhibited the cell viability and neoplastic transformation in (HSC-3 and TPA-treated JB6) cells by inducing apoptosis through ROS generation, and also increased the expression of cleaved PARP, Caspase-3, and Bax [151]. Moreover, α-mangostin extracted from *Garcinia mangostana* inhibited the growth and proliferation of human OSSC cells (HSC-2/-3/-4) by inducing apoptosis through a decrease in mitochondrial membrane potential and translocation of cytochrome c and also arrested cell cycle at G1 phase by downregulating (CDks/cyclins) [152]. Thymol isolated from thyme and oregano possesses potent antitumor activity and induces mitochondrial dysfunction and apoptosis in OSCC in vivo model [153]. Polyphenon E, in combination with erlotinib, has been used in clinical trials for the treatment of HSSCs (NCT01116336). Similarly, cucurbitacin B, in combination with other chemotherapeutic drugs, has shown antitumor activity against HSCC and breast cancer [154]. Several in vitro studies have shown that grape seed proanthocyanidins and EGCG produce cytotoxic effects by inducing apoptosis and cell cycle arrest at the G0/G1 phase in HSCC cell lines through various mechanisms [155].

### 3.8. Prostate Cancer

Prostate cancer is the most commonly diagnosed deadliest cancer in men after lung cancer in the world, having a mortality rate of 3.6% in 2018 and 1.05% increase is expected by 2040 [156]. The estimated rate of prostate cancer diagnosed in men is (one in nine) and mortality (1 in 41), which is quite alarming [157]. The etiological evidence of prostate cancer incidence, progression, and development is not very clear, and various risk factors such as age, race, family history, genetic/somatic mutations, and lifestyle have been linked to it. In particular, age has been a critical factor, and 66 years is the average age of diagnosis [158]. Like other cancers, early diagnosis and treatment are extremely important, but the asymptomatic nature of the disease and complicated diagnostic procedures and treatment expenses make it difficult to manage it properly. Different bioactive compounds have been evaluated and have shown potential to be used for the prevention and treatment of prostate cancer. For example, Shukla et al. tested apigenin against prostate cancer and found that the tumorigenesis in TRAMP mice was suppressed through inhibition of the Nf-kβ pathway [159]. Similarly, afzelin derived from *Nymphaea odoratum* exhibits anti-tumoral activities against prostate cancer (LNCaP and PC-3) cells by arresting cell cycle at G0 phase and also inhibits the expression of LIM domain kinase-1 [160]. Recently, plectronthoic acid, a novel compound, demonstrated potent significant anti-tumor activities by inhibiting proliferation, induced G0/G1 phase arrest in prostate cancer (PC3, DU145, and CW22Rv1) cells via upregulation of p21/CIP1 and p27/KIP1, and also suppressed mTOR/S6K signaling pathway [161]. In another study, anacardic acid has been shown to inhibit the proliferation and induce apoptosis in human LNCaP cells by autophagy through ER stress/DAPK3/Akt pathway [162]. Delphinidin induces p53 mediated apoptosis by suppressing HDAC function and activating acetylation of p53 in LNCap cells [163]. Moreover, lycorine extracted from Amaryllidaceae plants inhibited the growth, proliferation, migration, and invasion of multiple prostate cancer cells in vitro (LNCaP, PC-3M, DU145, and 22 RV1) and in vivo (PC-3M xenograft) model by abrogating p-STAT3 expression, reversed EMT via STAT3-mediated twist decrease, and inhibited EGF-induced JAK/STAT signaling [164].

Lall et al. established that fisetin effectively inhibits the synthesis of hyaluronan (HA) and increases antiangiogenic high molecular mass-HA and can be used to manage prostate cancer [165]. Similarly, punicalagin, a polyphenol, exhibited apoptotic activity and inhibited the proliferation of PC3 and LNCaP cells via upregulating caspase-3/-8 expressions and decreasing vascular network formation in the CAM model, which also confirmed its antiangiogenic effect [166]. Furthermore, Zeylenone isolated from *Uvaria grandiflora* Roxb decreased the cell viability, invasion, and metastatic growth of human PCa (DU145) cells by downregulating the Wnt/β-catenin pathway [167]. Moreover, mangiferin has been reported to possess immunomodulatory, apoptotic, antiangiogenic, and gene regulatory effects in vitro, ex vivo, and in vivo in different cancers, especially prostate cancer [168]. Similarly, anethole inhibited the proliferation of prostate cancer PC-3 cells and induced apoptosis by generating ROS, decreasing mitochondrial membrane potential, DNA damage, activating caspase-3 and -9, increasing Bax/Bcl-2 ratio, and leading to G2/M phase cell cycle arrest [169]. In addition, Paeonol inhibited tumor growth in vitro and in vivo by activating intrinsic and extrinsic apoptotic pathways and also inhibited p13k/Akt signaling pathway [170]. Similarly, oleandrin inhibits tumor progression by deregulating multiple pathways such as MAPK, Nf-kβ, and p13k/Akt pathway [171]. Ramamoorthy et al. reported that reserpine isolated from *Rauwolfia serpentine* induced apoptosis and arrested cell cycle at the G2 phase in prostate cancer (PC3) cells [172].

### 3.9. Hematological Cancer

Adverse events associated with the use of current therapies such as toxicities, neuropathy, or incessant relapsing have paved the way for using phytochemicals in the treatment of various hematological malignancies such as leukemia, lymphomas, multiple myelomas, Wald Enstrom’s macroglobulinemia, and other hematological diseases such as hemolytic anemia and thrombocytopenia. The frequently used natural products in hematological malignancies are anthracyclines and anthracenediones, vinca alkaloids, isothiocyanates, podophyllotoxin derivatives, polyphenols, and other antioxidants [173,174]. These phytochemicals are also used to sensitize conventional therapeutics such as dexamethasone. Curcumin, agaricus, and neovastat are currently in clinical trials for the treatment of multiple myelomas [175]. Vinca alkaloids such as vincristine and vinblastine are widely used as chemotherapeutic agents for various solid tumors and hematological disorders. Alkaloids such as harringtonine and isoharringtonine, which are isolated from *Cephalotaxus harringtonia*, act as chemotherapeutic agents and are used in the treatment of acute and chronic myelogenous leukemia and myelodysplastic syndromes. Isothiocyanates are another useful family of electrophilic bioactive compounds such as sinigrin, glucotropaeolin, gluconasturtiin, and glucoraphanin, and are involved in the inhibition of leukemic cells growth and regulate progression and differentiation of tumors, cell cycle, and apoptotic mechanisms. In a recent study, Wu et al. reported that rocaglamide breaks the TRAIL-induced resistance in vitro and in vivo in multiple myeloma and acute leukemia by inhibiting c-FLIP (main factor in TRAIL therapy resistance) expression and thereby increasing the effects of camptothecin, which shows that combined use of rocaglamide can be a suitable therapeutic option [176]. In addition, conophylline, an alkaloid isolated from *Tabernaemontana divaricata*, suppressed the pancreatic cancer desmoplasia and associated cytokines (IL-6, IL-8, CCL2, and CXCL12) produced by cancer-associated fibroblasts (CAF) and stellate cells. Moreover, the inhibitory and apoptotic effects of conophylline increased more when used in combination with gemcitabine [177]. Most recently, Tubulosine from *Alangium salvifolium* wang has selectively inhibited JAK3 signals by binding to ATP-binding active site of the kinase (JAK3), thereby reducing the progression and survival of hematopoietic cancer, as shown against HDLM-2, L540, U266, and BKO-84 cancer cells [178].

Similarly, betulinic acid has shown promising cytotoxic effects in various tumors. Phytochemicals provide a wide range of cellular effects by preventing carcinogens from reaching targeted sites and helping in ROS detoxification, increasing immunosurveillance to eliminate transformed cells, activating DNA repair mechanisms, and inhibiting proliferative tumor pathways [31]. In a recent study by Karami et al., gaillardin, a sesquiterpene lactone isolated from *Inula ocular-christi*, has been shown to produce higher cytotoxic effects in leukemic cells (NALM-6 and MOT-4 with IC50 of 6.1 and 7.3 µM, respectively) by arresting G0/G1 phase and inducing apoptosis, but it shows no cytotoxicity in normal cells [179]. Medicarpin, a natural phytoalexin, has been reported to sensitize myeloid leukemia cells to TRAIL treatment by upregulating pro-apoptotic markers (Cytochrome-c, tBid, Bax, CHOP) and downregulating anti-apoptotic proteins (Bcl-xl, Bcl-2, c-FLIP, XIAP, and survivin). Furthermore, it causes G2/M cell cycle arrest and also increases DR5 expression via activation of the ROS-JNK-CHOP signaling pathway [180]. Furthermore, taxodione isolated from *taxodium distichum* has been found to induce apoptosis in leukemia k562 cancer cells by generating ROS. Moreover, BCR-ABL, Akt, and STAT5 are sequestered in the mitochondria and are unable to stimulate proliferation [181].

### 3.10. Miscellaneous Cancer

Many different types of cancers have been affecting and threatening the lives of people, which include skin, stomach, oral cavity, rectum, gastric, gallbladder, pancreas, cervix uteri, penis, kidney, bladder, thyroid, Hodgkin lymphoma, non-Hodgkin lymphoma, leukemia, and osteosarcoma [182]. The interest in using natural products to treat cancer has been rising due to the better efficacy, low toxicity, and lower cost. Recently, Shivamadhu et al. showed that *praecetrullus fistulosus* lectins inhibited the growth and proliferation of a variety of tumor cells such as Hela, MCF-7, K562, and HT29 cells and also increased the survival of EAC bearing mice by decreasing MMP 2/9 activity and inducing apoptosis in the tumor cells [183]. In another study, oxolane derivatives from *Morus alba*, such as odisolane, have been proven to inhibit angiogenesis by reducing VEGF, p-ERK effectively, and p-Akt expressions, and can be employed to stop neovascularization in tumors [184]. Recently, hispiloscine and hispidacine alkaloids isolated from *Ficus hispida linn* showed excellent cytotoxic activity against various cell lines such as breast, lung, and colon, and also demonstrated vasorelaxant activity in rats [185]. Recently, Kim et al. demonstrated that bakuchiol inhibited the viability and EGF-induced neoplastic transformation of skin epithelial carcinoma A431 cell line in vitro and in vivo studies through the inhibition of Blk, Hck, p38MAPK, and Akt/p70S6k pathways [186]. Similarly, cryptolepine inhibited non-melanoma skin cancer proliferation by damaging DNA, S- phase cell cycle arrest, and decreasing membrane potential [187]. Curcubitacin B suppressed the invasion and proliferation of gastric cancer cells through STAT3 inhibition, which also induced apoptosis and furthermore in combination with cisplatin produced increased cytotoxic effects, which indicate that curcibitacin B is a promising STAT3 inhibitor [188]. Phycocyanin is another promising molecule isolated from seaweed, and it exerts its effects by arresting the cell cycle at the G2/M phase in MDA-MB-231, HT29, and A549 cells by decreasing cyclin E and CDk2 expressions and upregulating p21. Furthermore, it activates the mitochondrial apoptotic pathway while inhibiting the proliferative pathways such as PI3K/Akt/mTOR, MAPK, and Nf-kβ pathway [189].

In another study, Liang et al. demonstrated that isovitexin suppresses the stemness of osteosarcoma (U2OS and MG63) cells and induces apoptosis by disrupting the DNMT1/miR-34a/Bcl-2 nexus [190]. Furthermore, melatonin, a natural hormone produced by the pineal gland of animals, has been reported to inhibit almost all the hallmarks of tumor such as angiogenesis, metastasis, dysregulated metabolism, immune evasion, replicative immortality, and proliferative signaling, and induces apoptosis in various tumors an in vitro and in vivo studies [191]. Furthermore, purpurogallin inhibits the anchorage-dependent/independent growth of the esophageal squamous cell carcinoma in vitro and in vivo through the inhibition of MEK1/2 and ERK1/2 signaling pathways and, moreover, induces cell cycle arrest at S and G2 phases by decreasing Cyclin A1 and cyclin B2 and also activates PARP which induces apoptosis [192]. Xie et al. reported that mahanimbine inhibits the growth, proliferation, and viability of bladder cancer cells by inducing apoptosis with an increase in the expression of Bax and a decrease in Bcl2 levels, and also causes cell cycle arrest at Go/G1 phase. Furthermore it also induced autophagic death by increasing the LC3II and p62 expressions [193]. Similarly, taurine, an important amino acid present in different tissues of the body, has been proven to have antitumor effects in breast, lung, liver, colon, and prostate cancer by suppressing proliferation, invasion, and metastasis and inducing apoptosis [194]. Some of the common natural products which are undergoing different pre-clinical research are presented in Table 1.

## 4. Novel Formulations of Natural Products for Chemotherapy

The major concern of traditional therapies is their poor accessibility to the tumor site; therefore, higher doses are required to achieve a desired pharmacological response, which causes adverse drug reactions. The advancement of innovative nanotechnologies in medicines can significantly improve the treatment at clinical settings by overcoming the existing limitations associated with the diagnosis and treatment of various fatal diseases. Despite a wide range of antitumor effects exhibited by natural compounds in vitro preclinical studies, various hurdles still exist in translating these promising results into in vivo or clinical trials, resulting in failure and expectations. Despite remarkable health benefits, natural products’ full clinical potential has not yet been unlocked due to low aqueous solubility, poor absorption, and lower bioavailability, and shorter retention time in the biological environment. After administration, natural agents need to interact with various physico-chemical barriers that can alter their structure and affect their antitumoral activity. Therefore, novel formulation strategies are adopted to prevent the degradation of the natural compound and their parent structure, which helps retain their chemopreventive and chemotherapeutic activities [412].

Nanobased formulations in the size range of 30–100 nm have a higher surface area and can easily pass through the membranes and be absorbed. These unique characteristics of nanoparticles make them significantly attractive because it can easily overcome the inherent poor solubility and absorption problem of many natural compounds and other chemical entities. Various nano-based formulations have been employed for drug delivery in order to achieve targeted delivery, improve aqueous solubility and bioavailability, and also enhance the retention time, thereby minimizing the adverse effects and toxicities and also protecting the drug molecules from the detrimental effects of the bio environment such as enzymatic attack, pH fluctuation, and biochemical degradation [413]. Nanoencapsulation can protect and deliver the natural agents in their natural structural form and target it to the body’s specific tissues. Various nanostructures are designed and developed for drug delivery applications such as polymeric nanoparticles, liposomes, micelles, nanogels, solid lipid nanoparticles, dendrimers, nanocapsules, nanoemulsion, metallic, and ceramic nanoparticles.

Recently, Muosa et al. prepared ellagic acid (EA) or diindolylmethane (DIM) loaded PEG-PLGA nanoparticles, tested them against pancreatic cancer SUIT2-luciferase cells, and observed that the nanoformulation of the bioactive compounds produced more significant effects than the compounds alone by inhibiting the viability, angiogenesis, and growth of the pancreatic tumor cells [414]. Similarly, β-sterol loaded PLGA nanoparticles showed better antitumoral activity against breast cancer MDA-MB-231 and MCF-7 cell lines by inhibiting the proliferation and growth of the cells [415]. Recently, Feng et al. demonstrated that sequential delivery of α-mangostin and triptolide loaded polymeric micelles can improve the permeation and therapeutic efficacy in pancreatic cancer by inactivating cancer-associated fibroblast triggered by TGF-β and, as a result, can increase the perfusion at the tumor site, induce apoptosis, and inhibit proliferation in the orthotopic model of pancreatic ductal adenocarcinoma [416]. In a recent study, PEGylated betulinic acid loaded liposomes were developed and were shown to have better tumor inhibitory effects in in vitro (HepG2 and Hela cells) and in vivo (U14 cells xenograft) cancer models [417]. Recently, Liu et al. prepared a coordination nanoassembly of luteolin and ferric ions, which increased the solubility, stability, absorption, and efficacy, and acted as a chemotherapeutic agent as well as photothermal agent, which shows that it can overcome the problem of stability and increase its therapeutic efficacy [418].

A potential clinically effective therapeutic approach is the combinatorial delivery of bioactive molecules, producing synergistic or additive effects at lower doses, and minimizing the associated toxicities. Ahmadi et al. co-encapsulated hydroxystyrol and doxorubicin in PLGA-co-Acrylic acid nanoparticles and delivered them to HT-29 colon cancer cells, which resulted in higher apoptosis and cell cycle arrest and regulated the genes expression better than the single-loaded formulations and free drugs [419]. Similarly, the liquid crystalline nanoparticle of resveratrol and pemetrexed was developed for the management of lung cancer and tested against A549 lung cancer cells and urethane induced lung cancer model in mice, and the results indicated that the cytotoxicity, cellular uptake, and antitumoral activity was significantly improved compared to the drugs alone [420]. Furthermore, paclitaxel loaded artesunate-phospholipid liposomes have been tested against MCF-7, HepG2, and A549 cell lines, and it showed better cellular internalization and synergistically enhanced the in vitro antitumoral efficacy against all the tested cell lines [421]. Moreover, Wang et al. developed a hyaluronic acid-coated PEI-PLGA nanosystem for co-delivery of gambogic acid and pTRAIL for triple-negative breast cancer (TNBC) therapy. They observed that combinatorial nanosystem significantly inhibited tumor growth and proliferation by inducing apoptosis in vitro and in vivo tumor xenograft model of TNBC [422].

Similarly, Bian et al. investigated the combinatorial anticancer effects of curcumin and sorafenib-loaded lactosylated nanoparticles against the hepatocellular carcinoma HepG2 cell line HCC tumor xenograft model. They found that the combinatorial nanosystem even reduced the smallest tumor volume and exhibited the strongest inhibitory potential with lower systemic toxicity [423]. In addition, Wang et al. developed a novel PB@MIL-100 (Fe) dual metal-organic framework loaded with artemisinin for dual photothermal/chemotherapeutic purpose and evaluated in vitro and in vivo studies and found that the d-MOFs system produced synergistic effects with lower toxicity and can be a good option to be utilized as a photothermal-chemotherapeutic nanomedicine for the cancer therapy [424].

Nanocrystals are an important and emerging carrier system for delivering therapeutics. Recently, Wang et al. synthesized hyaluronic acid-coated camptothecin-loaded nanocrystals, evaluated them against various cancer cell lines such as MDA-MB-231, MCF-7, HepG2, and IMR-90 cells, and demonstrated that HA decorated CP nanocrystals improved the cytotoxicity, specificity, anti-migration activity, and antiproliferative activity [425]. Gupta et al. conjugated/encapsulated berberine in PAMAM dendrimers and evaluated them against MCF-7 and MDA-MB468 cell lines and in vivo pharmacokinetic studies in albino rats. They found that the conjugated berberine produced more significant and improved anticancer effects than the berberine encapsulated dendrimers [426]. Ding et al. reported a novel nanogel carrier system for co-delivery of EGCG and siRNA for drug-resistant breast cancer therapy and demonstrated that the multicomponent nanogel carrier has more pronounced cytotoxic effects and showed improved anticancer activity in in vitro MDA-MB231 cell line and in vivo tumor xenograft model [427]. Nanoparticles can specifically target cancer cells and enhance the specificity and efficacy of therapeutic modalities, resulting in improved patient compliance, response, and survival. However, the nanomaterial used for drug delivery applications must be biocompatible and biodegradable so that the unloaded material can be degraded into non-toxic metabolites and removed from the body via circulation. Various nanomaterials for cancer treatment are at the preliminary stages of research, and more significant in vivo data with excellent clinical efficacy are required in order to bring it to clinical settings for commercial use.

**Figure 4:**
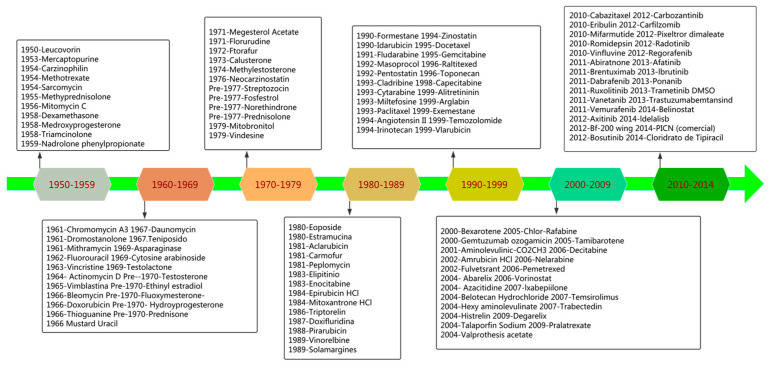
Approved marketed anticancer drugs from 1950 to 2014 which are directly obtained or derived from natural products or imitate them (adopted from [428], licensed under creative common attribution license).

Moreover, various new natural compounds of different structures, isolated from various plants, have been deemed as models, leads, or heads of series, and their structural alteration has resulted in compounds with pharmacological action and remarkable therapeutic potential outcomes (Figure 4). This research area, which is persistently growing and is of tremendous current interest, investigates new natural products originating from various sources. The significance of natural compounds in anticancer medication exploration could go past molecular diversity and novelty in structures (Table 2). The discovery of new natural compound structures with critical biological significance and new mechanisms of activity could likewise pioneer innovative research.

The increasing trend in natural products research shows that natural products have sustained their credibility as a viable and fertile area for drug discovery and advancement. Otherwise, there would have been a substantial therapeutic deficit in many most important clinical areas, such as cancer, cardiovascular disease, and neurodegenerative diseases. The most encouraging part is the continuous introduction of new natural product chemotypes with intriguing structures and biological activities, which are highly diverse and respond to much wider screening opportunities provided by a plethora of newly discovered targets. Natural products can provide a basic structural unit, and many lead molecules can be built upon by providing some useful input from the synthetic chemical libraries and utilizing modern computational and high throughput allied technologies.

## 5. Conclusions and Future Perspectives

In summary, the development of cancer is a complex process that is influenced by many factors. The present review highlights the importance of natural molecules in cancer management, since natural products provide inexhaustible sources of compounds with unique structures and new mechanisms of action. Moreover, natural compounds, either used alone or in combination, can be beneficial in the treatment and prevention of cancer. However, further studies are needed to describe the effect of natural compounds on cancer progression. In addition, it is evident that there is still much to be explored based on the natural diversity of the world, and because of technological advancements there are several new prototypes for pharmacologically active compounds appearing in screening programs, which will accelerate the exploration of natural compounds for different diseases such as cancer.

The growing incidence of cancer and limitations of conventional therapy such as higher costs, toxicities due to non-specific targeting, increased chemoresistance, and metastasis has led to tumor recurrence and posed a serious challenge to design and develop an alternative biocompatible, eco-friendly, and cost-effective strategy to combat cancer. Primary prevention is a better way to control cancer, benefiting both present and future generations, and estimates suggest that 30 to 50% of incident cancers can be prevented by translating prior knowledge of causes into effective interventions. Under this scenario, natural products are expected to revolutionize cancer treatment in the next decade due to their higher efficacy, biodegradability, and biocompatibility. Though the clinical applications of natural compounds are limited due to their poor aqueous solubility, rapid catabolism, targeting specificity, poor intestinal absorption, and reduced bioavailability, although nanotechnology-based formulations are used to overcome these limitations. Moreover, there is a lack of insight into the molecular interactions of natural compounds with various signaling molecules at the preclinical stage; for this purpose, in silico approaches such as molecular docking are needed to understand the interactions of natural products in different signaling pathways and identify their associated carcinogenesis biomarkers which can be further confirmed by many in vitro and in vivo models. Additionally, methodological flaws in various clinical studies such as smaller sample size, lack of placebo or control groups, and shorter trial duration have been observed. Thus, for many natural products, it is important to perform large-scale and well-controlled clinical trials to confirm and validate their anticancer activities, adverse effects, and safeties prior to their usage as anticancer drugs/adjuvants. Furthermore, extensive standardization based on modern approaches by evaluating their efficacy, composition, dosage-regimen, safety, quality, bioavailability, manufacturing practices, and regulatory and approval procedures need to be performed on the promising natural products to meet the quality and criteria of the international standard. Interestingly, pharmaceutical industries have vast experience and knowledge about drug development, and, therefore, combining the advantages of modern and traditional medicines can increase the development and availability of natural compounds for patient use.

A novel integrated approach for drug discovery, taking advantage of the ethnopharmacological knowledge validated and confirmed through the interdisciplinary efforts involving natural products chemistry, pharmacology, medicinal chemistry, biochemistry, cellular and molecular biology, is required to harvest the true potential of natural products. The higher biofunctionality and biodiversity of natural products make them attractive and unique from a drug discovery perspective, which needs to be further explored using modern screening techniques, which can provide various new prototypes that are pharmacologically active, thus increasing the speed of exploitation of natural compounds. Moreover, advancements in analytical techniques and computational chemistry coupled with artificial intelligence can help in the facilitation and identification of new natural active molecules for pharmacological evaluation. Further research on natural products and their limitations may lead to the discovery of new potent anticancer therapeutics with enhanced efficacy, safety, and quality for chemoprevention and chemotherapy.

In summary, this review is intended to increase scientists’ and researchers’ awareness of natural products’ diverse benefits, which can be utilized to develop safer and newer cancer treatments, as well as provide a solid foundation for future studies on natural compounds in cancer therapy. In the future, further studies are needed to investigate combinations of natural products comprising more than two natural products or combinations of existing chemotherapeutic drugs with less frequently utilized natural products. Moreover, many of these products were tested on limited cancer types, so their spectrum of activity should be expanded. Bioavailability limits the effectiveness of naturally occurring compounds. Thus, researchers must focus not only on the efficacy of the compound, which is of high interest, but also on drug delivery systems able to overcome pharmacokinetic issues, in addition to studying derivatives with a high degree of biological efficacy and availability.

## Figures and Tables

**Table 1:** Some of the important natural products in managing different types of cancers.

Sources	Bioactive Constituents	Chemical Formula	Molecular Weight (g/mol)	Mechanisms	Study Model	Concentrations	Other Cancers	References
**Lung cancer**								
*Fascaplysinopsis Bergquist*	**Fascaplysin**	C_18_H_11_ClN_2_O	306.7	↓CDK-4, ↑ROS, ↓mTOR, ↓4EBP1, ↓p70S6K1	A549, H1299, PC-9 and NCI-H526 cell lines	IC_50_: 0.57–1.15 µM	Liver, ovarian, and colon cancer	[195]
*Chelidonium majus*	Chelidonine	C_20_H_19_NO_5_	353.4	↓cyclin B1, ↑p21, ↓MMP, ↑ROS/AMPK, ↓p-p70S6K, ↓EGFR, G2/M phase arrest	A549, PC9, H460, H358 cells and H1975 xenograft in mice	IC_50_: 2.58–20 µM	Breast, liver, head, and neck cancer	[196]
*Cephaelis Ipecacuanha*	**Emetine**	C_29_H_40_N_2_O_4_	480.6	↓MMP-2/-9, ↓ERK1/2, and ↑p38	A549 and H1299 cell line	IC_50_: 0.243 and 1 µM	Breast, colon, prostate, and pancreatic cancer	[197]
*Citrus limon*	D-limonene	C_10_H_16_	136.23	↑Bax, ↓Bcl-2, ↑cleaved PARP, ↑Atg5	H1299 and A549 cells xenograft in Balb/c mice	0.5–0.75 mM	Skin, liver, breast, and kidney cancer	[198]
*Artemisia annua*	Sesquiterpene (AMDT)	C_17_H_26_O_2_	262.39	↑caspase 3- 9, G1/S phase arrest	95-D, HO8910, QGY, and HeLa cells	IC_50_: 52.44–73.3 µM	Gastric and ovarian cancer	[199]
	Artemisinin	C_15_H_22_O_5_	282.33	↓VEGF-C, ↓p38 MAPK	LLC cells and C57BL/6 mice	5–20 µM	Breast, cervical, gastric, liver, and bladder cancer	[200]
	Artemether	C_16_H_26_O_5_	298.37	↓Bcl-2, ↓cIAP1, ↓cIAP2, ↓CDK1, ↓CDK2, ↓CDK6, ↓cyclin A2, ↓cyclin B1, ↓cyclin D1, ↑p16	A549, NCI-H1299 cells	40–80 µM	Brain, breast, and gastric cancer	[201]
*Betula platyphylla*	Betulin	C_30_H_50_O_2_	442.7	↑caspase 3- 9 and 8, ↑ Bax, ↑Cyto- C, ↑Bak, ↓PARP	A549, HepG2, Hela, MCF-7 cells	IC_50_: 10–15 µg/mL	Gastric, renal, colon, and melanoma	[202]
	Betulinic Acid	C_30_H_48_O_3_	456.7	↓cyclin A2, ↓Sp1, G2/M phase arrest	A549, Hela, H1299 xenograft mice and *Scgb1a1-rtTA/TetO-Kras^G12D^*mice	≤40 µM	Colon, breast, prostate, nasopharyngeal, and cervical cancer	[203,204]
*Garcinia*	**Gambogic acid**	C_38_H_44_O_8_	628.7	↑Caspase-3, ↓Bcl2, ↓pI3K, *↓*DLL1, ↓DLL3, ↓ DLL4, ↓Jagged1, ↓Jagged2	SPC-A-1 and A549 cells	0.5–1 µmol/L	Liver and colorectal	[205]
*Laminaria japonica*	Fucoxanthin	C_42_H_58_O_6_	658.9	↑p21, ↑p53, ↑puma, ↑Fas, ↓Bcl-2, G0/G1 phase arrest	SPC-A1, A549, H460, H1299	IC_50_: <82 µM	Skin, gastric, breast, and colon cancer	[206]
*Chinese trichosanthes*	Trichosanthin	247 Amino acids	27 kDa	↑Caspase-3 and 9, ↑E-cadherin, ↓N-cadherin, ↓Bcl-2, ↑ Bax, ↑DR4, ↑DR5	NCI-H1299, NCI-H1975, and A549 cells	IC_20_: 39.5, 61.1 and 49.3 µg/mL	Cervical, gastric, breast, and nasopharyngeal	[207]
*Hedyotis*	**Ursolic acid**	C_30_H_48_O_3_	456.7	↓MMP2, ↓MMP 9, ↑E-cadherin, ↓N-cadherin	H1975 cells	IC_50_: 29.7 nM	Breast, prostate, cervical, colorectal, and liver cancer	[208]
	Oleanolic acid	C_30_H_48_O_3_	456.7	↑ROS, ↑Ca^2+^, ↓Akt, ↓JACk2, ↓STAT3	A549 cells	IC_50_: 9.66 µM	Gallbladder, prostate, and gastric cancer	[209]
*Scutellaria barbata*	Wogonin	C_16_H_12_O_5_	284.26	↑caspase-3, 8 and 9, ↑ROS	A427, BEAS-2B and A459 cells	50 µM	Breast, cervical, and nasopharyngeal carcinoma	[210]
	Luteolin	C_15_H_10_O_6_	286.24	↑miR-34a-5p, ↑p21, ↑p53, ↓MDM4, ↑caspase-3 and 9	A549, and H460 xenograft model	IC_50_: 40 µM	Breast, ovarian, gastric, and prostate cancer	[211]
*Polygonum cuspidatum*	Polydatin	C_20_H_22_O_8_	390.4	↓NLRP3, ↓ASC, ↓p-NF-κβ p65	A549 and H1299 cells	50 µM	Breast, hepatic, renal, and ovarian cancer	[212]
*Rheum officinale*	Emodin	C_15_H_10_O_5_	270.24	↑PPARγ, ↑IGFBP1, ↓Sp1	H1975, and A549 cells xenograft mice	50 µM	Renal and cervical cancer	[213]
*Rhizoma zedoariae*	β-elemene	C_15_H_24_	204.35	↑caspase-3, ↑ROS, ↑Cyto- C, ↑Bad, ↓Bcl-2	A549/DDP cells	20 and 40 µg/mL	Liver, gastric, and bladder cancer	[214]
*Chansu*	Bufalin	C_24_H_34_O_4_	386.5	↑caspase-3, ↓Akt, ↓ ERK ½, ↓VEGF, ↓cMyc, ↓NF-κβ, ↓p38 MAPK, G1/S phase arrest	A549, NCI-H460	2.5–10 µM	Prostate, liver, and ovarian cancer	[215,216]
*Platycodon grandiflorum*	Platycodin-D	C_57_H_92_O_28_	1225.3	↑Atg-3, ↑Atg-7, ↑Beclin-1, ↑LC3-II, ↓ p-Akt, ↓p-p70S6K, ↓p-4EBP1, ↓ ERK ½	NCI-H460 and A549 cells	5–30 µmol/L	Colon, leukemia, and breast cancer	[217]
*Salvia miltiorrhiza*	Tanshinone IIA (TSIIA)	C_19_H_18_O_3_	294.34	↑Cyto- C, ↓ Bax, ↓MMP, ↑caspase-3 and 9	A549 cells	IC_50_: 14.5 µM	Prostate, glioma, leukemia, and hepatoma	[218]
*Tripterygium wilfordii Hook*	**Celastrol**	C_29_H_38_O_4_	450.6	↓Bcl-2, ↑ Bax, ↓Akt, ↑Cyto- C, ↓PARP, ↑Fas, ↑FasL	A549 cells	IC_50_: 2.12 µM	Prostate, brain, and liver cancer	[219]
*Curcuma longa*	Curcumin	C_21_H_20_O_6_	368.4	↑ROS, ↓ΔΨ_m,_ ↓SOD1, ↑caspase-3 and 9, ↑ Bax, G2/M phase arrest	A549 and SPC-A1 cells	10–25 µM	Pancreatic, gastric, prostate, and colorectal cancer	[220,221]
*Cinnamomum cassia*	Cinnamaldehyde	C_9_H_8_O	132.16	↑caspase-3, ↑PARP, ↓Bcl-2,↑Bax,↓Bcl-xL, ↓HIF-1α, ↓β-catenin, ↓MMPs, ↑E-cadherin	NCI-H1299, YTMLC, A549 xenograft model	IC_50_: 10.5, 32, 41 µg/mL	oral squamous cell carcinoma and colorectal cancer	[222]
*Coix lacryma-jobi* L.	CP-1 polysaccharide	n.d.	n.d.	↑caspase-3 and 9, ↓ΔΨ_m,_ S phase arrest	A549 cells	10–300 µg/mL	Breast and colon cancer	[223]
*Ophiopogon japonicus*	Ophiopogonin B	C_39_H_62_O_12_	722.9	↑E-cadherin, ↓N-cadherin, ↓linc00668, ↑miR-432-5p	A549, 293T and THP-1 cells	5 and 10 µmol/L	Colon, breast, and gastric cancer	[224]
*Tetrastigma hemsleyanum*	Radix Tetrastigma Hemsleyani Flavone	C_27_H_30_O_10_	514.5	↓MMP 2 and 9, ↓TIMP-1, ↑TIMP-2	A549 cells	0.5–10 mg/mL	Liver, leukemia, and stomach cancer	[225]
*Panax notoginseng*	Trilinolein	C_57_H_98_O_6_	879.4	↓Bcl-2, ↑ Bax, ↑ROS, ↓PARP, ↓Akt, ↑caspase-3, ↑Cyto- C	A549, MKN-45 and A498 cells	25–100 µg/mL	Liver, colon, and gastric cancer	[226]
*Fucus vesiculosus*	Fucoidan	C_7_H_14_O_7_S	242.25	↑ROS, ↑ATF4, ↑CHOP, ↑TLR4	A549, CL1-5 and LLC-1 xenograft C57BL/6 mice	100–800 µg/mL	Breast, liver, and colon cancer	[227]
**Breast cancer**								
*Rabdosia rubescens*	Oridonin	C_20_H_28_O_6_	364.4	↓Jagged2, ↓Notch1-4	4T1 cells and xenograft BALB/c mice	0.1–10 mmol/L	Gastric, esophageal, prostate, and pancreatic cancer	[228]
*Panax notoginseng*	Panaxadiol saponins	C_30_H_52_O_3_	460.73	↓NLR, ↓MPO, ↓G-CSF, ↓PU.1, ↓C/EBPα, ↓EP1, ↓GATA 1 and 2	4T1-Luc and xenograft BALB/c mice	0.1–40 µM	Lung, liver, and colon cancer	[229]
*Ganoderma lucidum*	Ganodermanontriol	C_30_H_48_O_4_	472.7	↓u-PA, ↓CDC20, ↓survivin	MDA-MB-231 cells	IC_50_: 11.6 µM	Gastric, prostate, and colon cancer	[230]
	Polysaccharides	n.d.	n.d.	↑SOD, ↑CAT, ↑GPx, ↓IL-1β, ↓IL-6, ↓TNF-α	MDA-MB-231 cells, Wistar rats	0.5–3 mg/mL	Melanoma, cervical, and colorectal cancer	[231,232]
*Maclura pomifera*	Pomiferin	C_25_H_24_O_6_	420.5	↑CANX, ↑BCAP31, ↑Mn-SOD,↑ACOX1,↓BMP-7 ↓ID 2 and 3	MCF-7 and MCF-10A	IC_50_: 5.2 µM	Liver, cholangiocarcinoma, and colon cancer	[233]
*Chansu*	Bufadienolides	C_24_H_34_O_2_	354.52	↓STAT3/Mcl-1, ↓Bcl-xL, ↓PARP, ↑caspase-3	MDA-MB-231, MCF-10A and MCF-7 cells	0.1–0.5 µM	Liver, leukemia, lung, and gastric cancer	[234]
*Cimicifuga foetida*	**Actein**	C_37_H_56_O_11_	676.8	↓VEGF, ↓pERK, ↓pJNK, ↓CD34, ↓CXCR4	HMEC-1, 4T1 cells xenograft in mice	IC_50_: 0.065 µM	Gastric, bladder, and lung cancer	[235]
*Astragalus membranaceus*	Formononetin	C_16_H_12_O_4_	268.26	↓MMP 2, ↓MMP9, ↓PI3K, ↓Akt, ↑TIMP-1 and 2	MDA-MB-231 and 4T1 cells	2.5–160 µmol/L	Cervical, bladder, and gastric cancer	[236]
	Astragulus Polysaccharides	n.d.	n.d.	↓Bcl-2, ↑ Bax, ↑NO, ↑TNF-α, G1/S phase arrest	MCF-7 cells	100–1000 µg/mL	Liver, lung, and gastric cancer	[237]
*Narcissus* L. *bulb*	**Narciclasine**	C_14_H_13_NO_7_	307.25	↑AMPK-ULK1, ↓PRAS40	MDA-MB-231, MCF-7, MCF-10A, BT483, HCC1937 and xenograft in mice	IC_50_: 5–100 nM	Brain, liver, and hematological cancers	[238]
*Cimicifuga racemosa*	KHF16	C_37_H_58_O_10_	662.85	↓XIAP, Mcl-1, ↓Survivin, ↓Cyclin B1/D1, ↓NF-kβ	MDA-MB-231, MDA-MB-468 and SW527	IC_50_: <10 µM	Gastric, bladder, and lung cancer	[239]
*Pueraria lobata*	Puerarin	C_21_H_20_O_9_	416.4	↓NFκβ, ↑AMPK, ↑ACC, ↑GSK-3β, ↓CREB	MCF-7/Adr cells	20–100 µM	Bladder, liver, and lung cancer	[240]
*Gardenia jasminoides Ellis*	Genipin	C_11_H_14_O_5_	226.23	↓Bcl-2, ↑ Bax, ↑caspase-3, ↑JNK, ↑p38 MAPK	MDA-MB-231 cells	IC_50_: 327 µM	Gastric, multiple myeloma, and bladder cancer	[241]
*Tetradium ruticarpum*	Evodiamine	C_19_H_17_N_3_O	303.4	↓u-PA, ↓MMP 9, Bcl-2, ↑ Bax, ↓CD1, ↓CDK6, G0/G1 arrest	MDA-MB-231 cells and xenograft BALB/c mice	IC_50_: 90 µM	Bladder, colorectal, pancreatic, and melanoma	[242]
*Euphorbia prolifera Buch-Ham*	Myrsinol diterpene (J196-10-1)	n.d.	n.d.	↓MDR, ↓Pgp efflux, ↑ATP hydrolysis	MCF-7/Adr cells	0.39–50 µM	Gastric, colon and lung cancer	[243]
*Solanum nigrum* L.	α-Solanine	C_45_H_73_NO_15_	868.1	↑Bax, ↓Bcl-2, ↓Ψm	4T1 cells and xenograft BALB/c mice	IC_50_: 17 µM	Pancreatic, esophageal, and prostate cancer	[244]
*Melonidus suaveolens*	**Melosuavine I**	C_41_H_42_N_4_O_6_	686.79	↑caspase 3, ↓Bcl-2, ↑p53	BT549 cells	IC_50_: 0.89 µM	Lung, colon, and prostate cancer	[245]
*Cnidium monnieri* (L.) *Cusson.*	Osthole	C_15_H_16_O_3_	244.28	↑caspase 9 and 3, ↓PARP, ↑p53 and p23, ↓Cdk2 and CD1	MDA-MB 435 cells	20–100 µmol/L	Liver and lung cancer	[246]
*Ophiopogon japonicus*	Ophiopogonin D	C_44_H_70_O_16_	855	↑Caspase 9 and 8, ↓cyclin B1 and G2/M arrest	MCF-7 cells	12.5–50 µmol/L	Prostate and laryngeal carcinoma	[247]
*Panax ginseng C. A. Mey.*	Ginsenoside Rg5	C_42_H_70_O_12_	767	↑p53, p21 and p15, ↓CD1, CE2 and Cdk4, ↑caspase 6,7,8 and 9, ↓PARP	MCF-7 cells	25–100 µM	Leukemia, gastric, and cervical cancer	[248]
*Epimedium brevicornum*	Icariside II	C_27_H_30_O_10_	514.5	↑Cl-Caspase-9,8,7,3, ↑ Cl-PARP, ↑ Bax, ↑Bcl-xL, ↑BimL, ↑Fas, ↓ FasL, ↑FADD, ↓MMP, ↑Cyto- C, ↑AIF	MCF-7 and MDA-MB-231 cells	IC_50_: 72.73 and 97.14 µM	Lung, melanoma, epidermoid, and prostate cancer	[249]
**Ovarian cancer**								
*Tripterygium wilfordii Hook*	**Triptolide**	C_20_H_24_O_6_	360.4	↓ MMP7 and MMP19, ↑Ecadherin	SKOV3, A2780 cells and SKVO3 xenograft mice	1.5–150 nM	Colon, renal, and cervical cancer	[250]
*Panax ginseng*	Ginsenoside 20(S)-Rg3	C_42_H_72_O_13_	785	↑ Caspase-3 and 9, ↓ PI3K/Akt and IAP	HO-8910 cells	25–100 µg/mL	Lung, prostate, and breast cancer	[251]
*Syzygium aromaticum*	Kumatakenin	C_17_H_14_O_6_	314.29	↑ Caspase-3,8 and 9, ↓MCP-1, ↓RANTES, ↓IL-10, ↓VEGF, ↓MMP 2 and 9	SKOV3 and A2780 cells	<100 µg/mL	Breast, liver, colon, and gastric cancer	[252]
*Pueraria mirifica*	Daidzein	C_15_H_10_O_4_	254.24	↑Bcl-2, ↑cym-c, ↑cleaved caspase-3/9, G2/M cell arrest, ↓pCdc25c, ↓pCdc2, ↓cyclin B1, ↓MMP-2/9	SKOV3 cell line	IC_50_: 20 µM	Breast, colon, bladder, and pancreatic cancer	[253]
*Thelypteris torresiana (Gaud)*	**Protoapigenone**	C_15_H_10_O_6_	286.24	↓p-Cdk2, ↓Cdk2, ↓p-Cyclin B1, ↓Cyclin B1, ↑p-Cdc25C, ↓Bcl-xL, ↓Bcl-2, ↑caspase-3	MDAH-2774 and SKOV3 cells	IC_50_: 0.69 and 0.78 µM	Lung, breast, and prostate cancer	[254]
*Epimedium brevicornum*	Icariin	C_33_H_40_O_15_	676.7	↑Caspase-3, ↓miR-21, ↑PTEN and RECK, ↓Bcl-2	A2780 cells	13–100 µM	Esophageal, prostate, and liver cancer	[255]
	Icaritin	C_21_H_20_O_6_	368.4	↑caspase 3- 9, ↑p53 and ↓Akt/mTOR pathway	OV2002, C13*, A2780cp and PDXs in NOD/SCID mice	10–50 µM	Endometrial, bladder, colorectal, and prostate cancer	[256]
*Gundelia tournefortii*	Stigmasterol	C_29_H_48_O	412.7	↑ROS, ↑caspase 3 and 9, Bax, ↑BAk, ↑cym-c, ↓VEGFA, ↓MMP 2,9 and 14	OV90 and ES2 cells	5–20 µg/mL	Gastric, skin, liver, and lung cancer	[257]
*Vitex Agnus-castus* L.	Casticin	C_19_H_18_O_8_	374.3	↑FOXO3a, ↓FoxM1, ↓survivin, ↓PLK1, p27KLP1	SKOV3 and A2780 cells	2.5–10 µmol/L	Gastric, gallbladder, cervical, and melanoma	[258]
*Carya cathayensis Sarg*	Juglone	C_10_H_6_O_3_	174.15	↑ROS, ↑p21, ↑Bax, ↑Bad, ↑Cyto c, ↓CDK2, cdc25A, CHK1, and cyclin A, ↓Bcl-2 and Bcl-xL, ↓Cyclin A, S-phase cell cycle arrest	Ishikawa cells	IC_50_: 20.81 µM	Glioma, lung, and leukemia	[259]
*Scutellaria baicalensis Georgi*	Baicalin and Baicalein	C_21_H_18_O_11_, C_15_H_10_O_5_	446.4, 270.24	↓VEGF, HIF-1α, cMyc, and NFκB	OVCAR-3, IOSE-364, and CP-70 cells	5–160 µM	Burkett lymphoma, colorectal, pancreatic, prostate, and osteosarcoma	[260]
*Potamogeton crispus* L.	Luteolin-3’-O-β-D-glucopyranoside	C_21_H_20_O_11_	448.4	↓MMP-2, ↓MMP-9, G1/S phase arrest	ES-2 cells	15–240 µg/mL	Colon and breast cancer	[261]
*Artemisia annua*	artesunate	C_19_H_28_O_8_	384.4	↑ROS, ↑p21, ↓CDKs, ↓Rb, ↓E2F-1, ↓CDC25C, G2/M cell cycle arrest	HEY, IGROV-1, OVCAR8, and OVCAR3 cells, and ID8 xenograft in C57BL/6 mice	IC_50_: 0.51–31.89 µM	Leukemia, pancreatic, and breast cancer	[262]
*Arctium lappa*	Arctigenin	C_21_H_24_O_6_	372.4	↓STAT3, ↓survivin, ↓iNOS, ↑caspase3	OVCAR3 and SKOV3 cells	IC_50_: 10 µM	Breast, colon, and lung cancer	[263]
*Asparagus officinalis* L.	Asparanin A	C_39_H_64_O_13_	740.9	↓Bak/Bcl-xl, ↑ROS, ↑Cyto c, ↓Δψm, ↑caspases, G0/G1 cell cycle arrest, ↓PI3K/Akt/mTOR	Ishikawa cells and xenograft BALB/c mice	IC_50_: 9.34 µM	Liver and pancreatic cancer	[264]
*Salvia miltiorrhiza*	Cryptotanshinone	C_19_H_20_O_3_	296.4	↑caspase3 and 9, ↑Bax/Bcl-2, ↓MMP 2 and 9	A2780 cells	5–30 µM	Leukemia, prostate, and colon cancer	[265]
**Colon cancer**								
*Asiatic Moonseed*	Dauricine	C_38_H_44_N_2_O_6_	624.8	↓cyclin D1, ↓COX2, ↓cMyc, ↓survivin, ↓Bcl-2, ↓IAP1, ↓MMP 9, ↓ICAM1, ↓VEGF, ↓NFκβ	HCT116, HCT8, SW480 and SW260 cells	5–20 µM	Pancreatic and renal cell carcinoma	[266]
*Withania somnifera*	Withaferin A	C_28_H_38_O_6_	470.6	↑ROS, ↓ Bcl-2/Bax, ↑caspase3 and 9, ↓ΔΨ_m_	RKO and HCT116 cells	0.1–10 µM	Lung, breast, and pancreatic cancer	[267]
*Piper nigrum*	Piperine	C_17_H_19_NO_3_	285.34	↓wnt/β-catenin pathway	SW480 and SW480-pBAR/*Renilla*, HCT116, DLD1, RKO	30–100 µM	Lung, liver, breast, and brain cancer	[268]
*Crocus sativus*	Crocetin	C_20_H_24_O_4_	328.4	↑p53, ↑PIDD, ↑Bax, ↑FAS, ↑caspase-3, -8 and -9	HCT116 and HT29 cell lines	100 µM	Prostate, breast, pancreatic, and gastric cancer	[269]
*Cynanchum paniculatum*	**Antofine**	C_23_H_25_NO_3_	363.4	↓proliferation, ↑cytotoxicity, G2/M cycle arrest	Col2 and A549 cells	IC_50_: <9 ng/mL	Breast, lung, and renal carcinoma	[270]
*Zingiber zerumbet*	Zerumbone	C_15_H_22_O	218.33	↑ROS, ↓ Bcl-2/Bax, ↑caspase-3/-8/-9, ↓ΔΨ_m,_ G2/M cycle arrest	SW480 cell line	IC_50_: 102 µM	Oral, breast, lung, and prostate cancer	[271]
*Hymenocallis littoralis*	Pancratistatin	C_14_H_15_NO_8_	325.27	↑LC3II, ↑beclin-1, ↑Bax, ↓cyclin B1, ↓cdc25c, G2/M cycle arrest	SW948, DLD1, HTC15, and HT29 cells	IC_50_: 15–25 µM	Lymphoma, breast, liver, skin, and teratocarcinoma	[272]
*Saussurea lappa*	**Costunolide**	C_15_H_20_O_2_	232.32	↓Survivin, ↓β-catenin, ↓galectin-3, ↓cyclin D1, G2/M cycle arrest	SW480, L-Wnt3a cells	0.5–5 µM	Breast, prostate, and ovarian cancer	[273]
*Rehmannia glutinosa*	Catalpol	C_15_H_22_O_10_	362.33	↓VEGF, ↓EGFR2, ↓HIF-1α, ↓IL-1β, ↓IL-6 and 8, ↓iNOS	CT26 cells and xenograft in mice	<40 µM	Gastric, lung, and liver cancer	[274]
*Laminaria japonica*	LJGP	n.d.	n.d.	↓CDK2, ↓PCNA, ↓E2F-1, ↓cyclin E, ↓cyclin D1, ↓PARP, ↑p27, ↑caspase 9, ↓Bcl-2, G1 phase arrest	HT-29, HepG2 and AGS cells	IC_50_: 100 µg/mL	Lung, liver, and cervical carcinoma	[275]
*Rosmarinus officinalis*	Rosmarinic acid	C_18_H_16_O_8_	360.3	↑E-cadherin, ↓N-cadherin, ↓twist, ↓vimentin, ↓MMP 2 and 9, ↓ICAM-1, ↓ITGβ1	CT26 and HCT116 cells	50–200 µM	Breast, gastric, leukemia, and cervical cancer	[276]
*Coptis chinensis*	Berberine	C_20_H_18_NO_4_^+^	336.4	↑p21, ↓PARP, ↑caspase 8, ↓VEGF, ↓COX2, ↓Bcl-2, G2/M phase arrest	SW480 cells	0.5–50 µM	Prostate, cervical, esophageal, thyroid, and gastric cancer	[277]
*Sanguinaria canadensis*	**Sanguinarine**	C_20_H_14_NO_4_^+^	332.3	↑ROS, ↓MMP, ↑caspase-3, 8 and 9, ↓Bcl-2, ↓XIAP, ↑Egr-1	HCT-116 cell line	0.3–1.2 µM	Breast, prostate, cervical, and pancreatic cancer	[278]
*Nauclea orientalis*	Naucleaoral A and B	C_20_H_20_N_2_O_3_	336.38	↑cytotoxicity	Hela and KB cells	IC_50_: 4.0 and 7.8 µg/mL	Cervical, bladder, and pancreatic cancer	[279]
*Glycyrrhiza uralensis*	Licoricidin	C_26_H_32_O_5_	424.5	↑caspase-3, 8 and 9, ↓CDK1, ↑AMPK, ↓Akt/mTOR, G1/S phase arrest	SW480 cells and xenograft BALB/c mice	IC_50_: 7.2 µM	Gastric, lung, prostate, and osteosarcoma	[280]
*Carpobrotus edulis*	Rutin	C_27_H_30_O_16_	610.5	↑caspase 3, G0/G1 phase arrest	HCT116 and HaCaT cells	1000 µM IC_50_: 679 µM	Breast, lung, and cervical cancer	[281]
*Scutellaria baicalensis*	Baicalin and Baicalein	C_21_H_18_O_11_, C_15_H_10_O_5_	446.4, 270.24	↓hTERT, ↓MAPK, ↓ERK, ↑p38	HT-29, SW480 cells and HCT-116 xenograft NSG mice	10–150 µM	Breast, prostate, and pancreatic cancer	[282]
*Nicotiana glauca*	**Scopoletin**	C_10_H_8_O_4_	192.17	↓ERK1, ↓VEGF-A, ↓FGF-2	HUVEC, CCD18-Co and HCT116 Xenograft mice	IC_50_: 0.06 µM	Breast, lung, and skin cancer	[283]
*Morus australis*	Morusin	C_25_H_24_O_6_	420.5	↓ PI3K/Akt, ↓PDK1, ↓XIAP, ↓cMyc, ↓NFκβ, ↑caspase-3, 8 and 9, G1 phase arrest	HT-29 cells	IC_50_: 6.1–12.7 µM	Breast, ovarian, and prostate cancer	[284]
*Allium sativum*	Diallyl disulphide	C_6_H_10_S_2_	146.3	↓GSK3β, ↓NFκβ	SW480 cells and AOM/DSS mouse model	2.5–40 µM	Lung, breast, and gastric cancer	[285]
*Nandina domestica*	Protopine	C_20_H_19_NO_5_	353.4	↑p53, ↑p21, ↑Bax, ↑caspase-3 and 7, ↑LC3-II	HCT116 cells	10–40 µM	Prostate, breast, ovarian, and head and neck cancer	[286]
**Brain cancer**								
*Nigella sativa*	Thymoquinone	C_10_H_12_O_2_	164.2	↑LC3-II, ↑p62, ↓MMP 2 and 9, ↓FAC, ↓Nf-kβ, ↓ERK, ↓Akt, ↓mTOR	T98MG and U87MG cells	10–40 µM IC50: 10.3 µM and 8.3 µM	Breast, liver, colon, and lung cancer	[287,288]
*Radix Angelica sinensis*	Z-ligustilide	C_12_H_14_O_2_	190.24	↓RhoA, ↓Cdc42, ↓Rac1	T98MG cells	2.5–25 µM	Breast, prostate, and colon cancer	[289]
*Panax ginseng C. A. Mey*	Ginsenoside Rh2	C_36_H_62_O_8_	622.9	↑miR128, ↑E2F3a, ↑caspase-3	T98MG, A172, and U251 cells	12 µg/mL	Breast, ovarian, colon, and prostate cancer	[290]
*Bolbostemma paniculatum*	Tubeimoside-1	C_65_H_102_O_29_	1347.5	↓Bcl-2, ↑ Bax, ↑caspase-3, ↑Cyto- C, ↑ROS	U251 and U87 cells	10–50 µg/mL	Gastric, liver, ovarian, and lung cancer	[291]
*Thuja occidentalis*	α-/β-Thujone	C_10_H_16_O	152.23	↓VEGF, ↓Ang-4, ↓CD31	U87-MG and C6 cells xenograft in mice	LD_50_: 400 and 300 µg/mL	Melanoma, breast, lung, and colon cancer	[292]
*Rubia cordifolia L.*	Mollugin	C_17_H_16_O_4_	284.31	↓ Akt, ↓ P70S6K, ↓ mTOR, ↓ ERK ½, ↑JNK, ↑p38	U87MG, U251 and MKN45 cells	10–40 µM	Breast, colon, ovarian, and lung cancer	[293]
*Garcinia brasiliensis*	7-epiclusianone	C_33_H_42_O_4_	502.7	↓cyclin A, ↑caspase-3, S and G2/M phase arrest	U251MG and U138MG cell lines	IC_50_: 23 and 18.52 µM	Colon, melanoma, breast, lung, and ovarian cancer	[294]
*Escherichia coli*	Selenocysteine	C_3_H_6_NO_2_Se	167.06	↑ROS, ↑p21waf1/cip1, ↑p53, ↓Akt, ↑p38MAPK, ↑JNK, ↑ERK, S phase cycle arrest	U251 and U87 cell lines	5–20 µM	Breast, lung, and prostate cancer	[295]
*Anula helenium*	Alantolactone	C_15_H_20_O_2_	232.32	↑ROS, ↓GSH, ↓Bcl-2, ↑ Bax, ↑p53, ↑Cyto- C, ↑caspase-3/-9, ↓ΔΨ_m_, ↓Nf-kβ	U87, U373 and LN229 cells	IC_50_: 33–36 µM	Lung, breast, liver, and pancreatic cancer	[296]
*Carpesium nepalense*	Nepalolide A	C_20_H_28_O_6_	364.4	↓IkB-α, ↓IkB-β, ↓iNOS, ↓NF-kβ	C6 cell line	2–10 µM	Brain cancer	[297]
*Buxus microphylla*	Cyclovirobuxine D	C_26_H_46_N_2_O	402.7	↑ Bax, ↓Bcl-2, ↑caspase-3, S and G0/G1 phase arrest	T98G and Hs683 cell lines	15–240 µmol/L	Gastric, colon, breast, and prostate cancer	[298]
*Euscaphis japonica*	Pomolic acid	C_30_H_48_O_4_	472.7	↑caspase-3 and 9, ↑ROS, ↓MRP1	A172, GBM-1 and U87 cells	IC_50_: 8.82, 9.72 and 11.09 µg/mL	Leukemia, melanoma, gastric and uterine cancer	[299]
*Danshen*	Salvianolic Acid B	C_36_H_30_O_16_	718.6	↑ROS, ↑p53, ↑p38 MAPK	U87 cell line	1–100 µM	HNSCC, breast, and colon cancer	[300]
*Glycine max*	Soyasapogenol B	C_30_H_50_O_3_	458.7	↓STAT3, ↓M2 polarization, ↑M1, ↑IL-12	U373-MG, SaOS2, and LM8 cells xenograft in mice	1–100 µM	Gastric, breast, colon, and renal cancer	[301]
*Rosmarinus officinalis*	Carnosol	C_20_H_26_O_4_	330.4	↑p53, ↓twist, ↓Zeb1, ↓slug, ↑miR-200c	U87MG, T98G, U373 MG	IC50: <40 µM	Lymphoma, lung osteosarcoma, and gastric cancer	[302,303]
*Tillandsia recurvata*	**HLBT-100**	C_19_H_20_O_8_	376.4	↑caspase-3, and 7, G1 cell cycle arrest, ↓angiogenesis	NCI60 cell lines (U87-cells)	GI50 values: <0.100 µM IC50 for U87: 0.054 µM	Breast, prostate, leukemia, and melanoma	[304]
*Ligusticum chuanxiong hort*	Tetramethylpyrazine	C_8_H_12_N_2_	136.19	↓CXCR4	C6 cell line	100 µM	Breast, liver, colon, and lung cancer	[305]
*Garcinia hanburyi Hook. f*	**Gambogenic acid**	C_38_H_46_O_8_	630.8	↓cyclin E, ↓cyclin D1, ↓EGFR, ↓Akt, ↓GSK3β, Go/G1 phase arrest	U251 cell line	0.75–6 µM	Gastric, breast, and lung cancer	[306]
*Vitis vinifera*	Resveratrol	C_14_H_12_O_3_	228.24	↓Akt, ↑p53	U87 and patient derived (22,33 and 44 GSC) cells	5–100 µM	Colon, gastric, and breast cancer	[307]
*Curcuma aromatica Salisb.*	Germacrone	C_15_H_22_O	218.33	↑p53, ↑ Bax, ↓Bcl-2, ↑p21, ↓CD1, ↓CDK2, G1 phase arrest	U87 and U251 cells	50–250 µmol/L	Breast, prostate, and liver cancer	[308]
*Acori Graminei Rhizoma*	Volatile Oil (VOA)	n.d.	n.d.	↑caspase-3, 8 and 9, ↑ Bax/Bcl-2, ↑LC3-II/I, ↑atg5, ↑beclin1, ↓p62	U87, U251, 3T3 and A172 cells	25–250 µg/mL	Pancreatic and breast cancer	[309]
*Trichosanthes kirilowii Maxim.*	Trichosanthin	247 Amino acids	27 kDa	↓LGR5, ↓β-catenin, ↓pGSK-3β^Ser9^, ↓cMyc, ↓CD1	U87 and U251 cells	IC_50_: 40 and 51.6 µM	Leukemia and cervical cancer	[310]
*Streptomyces staurosporeus*	**Staurosporine**	C_28_H_26_N_4_O_3_	466.5	↑caspase-3, ↓TDP-43	U87 cell line	IC_50_: 5µM	Lung, breast, prostate, and colon cancer	[311]
*Dysosma versipellis*	**Deoxypodophyllotoxin**	C_22_H_22_O_7_	398.4	↓Cdc2, ↓CB1, ↓Cdc25C, ↑caspase-8 and 9, ↓Bcl-2, ↓Bcl-xL, G2/M phase arrest	U-87 MG and SF126 cells	IC_50_: 15.06 and 13.95 nM	Lung, breast, prostate, and colon cancer	[312]
*Anemone taipaiensis*	**Saponin B**	C_48_H_78_O_17_	927.1	↑Fas-l, ↑caspase-3, ↓Bcl-2, G1/S phase arrest	U87MG cells	IC_50_: 6.7 µmol/L	Leukemia and breast cancer	[313]
**Liver cancer**								
*Strychnos nux-vomica Linn*	Brucine	C_23_H_26_N_2_O_4_	394.5	↓HIF-1, ↓MMP-2, ↓FN, ↓LOX, ↓CD	SMMC-7721, HepG2, and HCC in Male Kunming mice	20–150 µM	Colon and breast cancer	[314]
*Azadirachta indica*	**Nimbolide**	C_27_H_30_O_7_	466.5	↑caspase-3,7 and 9, ↑ Bax, ↓Bcl-2, ↓Mcl-1, ↓XIAP, ↓ c-IAP1, ↓c-IAP2, G2/M phase arrest	PLC/PRF/5 and Huh-7 cells xenograft Balb/c mice	1–5 µM	Pancreatic, breast, lung, and colon cancer	[315]
*Gardenia jasminoides ellis*	Geniposide	C_17_H_24_O_10_	388.4	↓miR-224, ↓wnt/βcatenin, ↓Akt	HepG2 and Huh7 cells	100–500 µM	Brain, oral, skin, and colon cancer	[316]
*Solanum nigrum* L.	Solamargine	C_45_H_73_NO_15_	868.1	↓pcna, ↓Ki67↓Bcl-2, ↑ Bax, ↑caspase-3 and 9, G2/M phase arrest	SMMC-7721 and HepG2 cells	IC_50_: 12.17 and 20 µM	Colon, lung, prostate, and breast cancer	[317]
*Matricaria recutita*	α- bisabolol	C_15_H_26_O	222.37	↑caspase-3,-8 and -9, ↑Fas, ↓Bcl-2, ↑p53, ↑Nf-kβ,	HepG2, ECa109, PC-3 and Hela cells	1–20 µM	Colon, brain, endometrial, and prostate cancer	[318]
*Ganoderma lucidum*	Ganoderic acid A	C_30_H_44_O_7_	516.7	↓cyclin D1, ↑p21, ↑cleaved caspase 3, G0/G1 phase arrest	HepG2 and SMMC-7721 cells	IC50: 187.6 and 158.9 µmol/L	Prostate, breast, lung, and meningioma	[319]
*Thalictrum glandulosissimum*	Hernandezine	C_39_H_44_N_2_O_7_	652.8	↑AMPK, ↑Atg-7	HepG2 and Hep3B cells	IC50: 7.42 and 6.71 µM	Lung, prostate, breast, and cervical cancer	[320]
*Angelica gigas Nakai*	Decursin	C_19_H_20_O_5_	328.4	↑LATS1, ↑βTRCP, ↑p-YAP, ↑cleaved caspase 3, ↑cleaved PARP, G1 phase cell cycle arrest	HepG2, Huh-7 cells and tumor xenograft in mice	5–80 µM	Gastric, lung, prostate, and lymphoma	[321]
*Lavandula officinalis*	Linalool	C_10_H_18_O	154.25	↓cyclin A, ↓CDK4, ↑p21, ↑p27, ↑ROS, ↑caspase-3, ↓Ras, ↓Akt, ↓mTOR, G0/G1 phase arrest	HepG2 cell line	0–2.5 mM	Leukemia, breast, prostate, and ovarian cancer	[322]
*Tylophora indica*	**Tylophorine**	C_24_H_27_NO_4_	393.5	↓cyclin A2, G1 phase cell cycle arrest	HepG2, HONE-1, and NUGC-3 cells	2 µM	Breast, stomach, nasopharyngeal, and colon cancer	[323]
*Patrinia scabra Bunge*	Lariciresinol	C_20_H_24_O_6_	360.4	↓ΔΨ_m_, ↑Cyto- C, ↑caspase-3 and 9, ↑PARP, ↓Bcl-2/Bax	HepG2 cells	IC50: 208 µg/mL	Leukemia, breast, and prostate cancer	[324]
*Oroxylum indicum*	**Oroxin B**	C_27_H_30_O_15_	594.5	↑PTEN, ↓COX-2, ↓VEGF, ↓p-Akt, ↓PI3K	SMMC-7721	0.34–1.68 µM	Breast, lung, and lymphoma	[325]
*Stephania tetrandra*	**Tetrandrine**	C_38_H_42_N_2_O_6_	622.7	↓wnt/β-catenin, ↓MTA1, ↑E-cadherin, ↑occludin, ↓Vimentin	Huh7, Hep3B and HCCLM9 xenograft Balb/c mice	0.5–4 µM	Colon, esophageal, and pancreatic cancer	[326]
*Scutellaria lateriflora*	Scutellarein	C_15_H_10_O_6_	286.24	↓HIF-1α, ↓Flt-1, ↓VEGFA, ↓MMP 2 and 9, ↑caspase-3	HepG2, MCF-7, EAC, A549 and liver carcinoma and ascites lymphoma model in mice	IC50: <13.8 µM	Gastric, colon, lung, and fibrosarcoma	[327]
*Toddalia asiatica Lam*	**Chelerythrine**	C_21_H_18_NO_4_^+^	348.4	↓MMP 2 and 9, ↓p-FAK ↓PI3K, ↓Akt, ↓mTOR, ↓c-JNK, ↓ERK	Hep3B Cell line	0.625–5 µM	Breast, prostate, renal, and lung cancer	[328]
*Astragalus complanatus R.Br.*	(FAC) flavonoids	n.d.	n.d.	↑caspase-3 and 8, ↑ Bax, ↑p21, ↑p27, ↓CDK1, CDK4, ↓cyclin B1, ↓cyclin D1, G0/G1 and S phase arrest	SMMC-7721 and HepG2 cells	IC_50_: 48 and 53 µg/mL	Breast and nasopharyngeal	[329]
*Quercus Iberica*	Quercetin	C_15_H_10_O_7_	302.23	↑E-cadherin, ↓MMP9, ↑LC3, ↓Vimentin, ↓Jak2, ↓STAT3,	LM3 cells and xenograft mice model	20–200 µM	Breast, colon, ovarian, and pancreatic cancer	[330]
*Trichosanthis Radix*	**Cucurbitacin B**	C_32_H_46_O_8_	558.7	↓cdc2, ↓cyclin D1, ↓c-Raf, S phase arrest	BEL-7402 cells and xenograft mice	IC_50_: 0.32 µM	Brest, pancreatic, and laryngeal cancer	[331]
*Colchium speciosum*	**Colchicine**	C_22_H_25_NO_6_	399.4	↑AKAP12, ↑TGFB2, ↑MX1, ↓APOH, ↑GDF15, ↑IL32	HCC24 and HCC38/KMUH, F28 and F59/KMUH cell lines and Balb/c-nu xenograft	2 and 6 ng/mL	Thyroid, oropharyngeal, and breast cancer	[332]
**Head and Neck cancer**								
*Wilkstroemia elliptica Merr.*	Umbelliferone	C_9_H_6_O_3_	162.14	↑ROS, ↓MMP, G0/G1 cycle arrest	HOC KB cells	IC_50_: 200 µM	Renal prostate, lung, and breast cancer	[333]
*Dioscorea nipponica*	**Dioscin**	C_45_H_72_O_16_	869	↑p53, ↓Cyclin A, ↓CDK2, ↓p-ERK, ↓Bcl-2, ↑p-JNK, ↑p-p38, ↑Bax, ↑cleaved caspase-3/-9	NP69, Hep-2 and TU-212 cells	IC_50_: <5 µg/mL	Gastric, breast, lung, ovarian, and colorectal cancer	[334]
*Maclura pomifera*	**Osajin**	C_25_H_24_O_5_	404.5	↑Bax, ↓Bcl-2, ↑Fasl, ↑cym-c, ↓GRP78, ↑caspase-3,4,8 and -9	TW076, TW04 and CG-1 cells	IC_50_: 5 µM	Kidney, prostate, breast, and colon cancer	[335]
*Cichorium intybus*	**Esculetin**	C_9_H_6_O_4_	178.14	↓pJAK1/2, ↑ROS, ↓STAT3, G1/S cycle arrest	TU-212, M4e, Hep-2 xenograft in mice	IC_50_: 2.969, 12.88 and 1.958 µM	Pancreatic, prostate, colon, and lung cancer	[336]
*Serratia marcescens*	**Prodigiosin**	C_20_H_25_N_3_O	323.4	↓Cyclin D1, ↑beclin-1, ↓mTOR, ↓PI3K/Akt, G0/G1 phase arrest	OECM1 and SAS cell lines	IC_50_: 1.59 and 3.25 µM	Breast, gastric, colon, and hematopoietic cancer	[337]
*Albatrellus confluens*	Neoalbaconol	C_22_H_34_O_3_	346.5	↓PDK1, ↓PI3K/AKT HK-2, ↑RIP1, ↑RIP3	NP69, k562,MCF-7 and A549 and C666-1 xenograft in mice	IC_50_: ≤18 µM	Lung, breast, colon, and gastric cancer	[338]
*Haematococcus pluvilis*	Astaxanthin	C_40_H_52_O_4_	596.8	↓PI3k/Akt, ↓STAT3, ↓Nf-kβ, ↓miR-21, ↓HOTAIR	SCC131 and SCC4 cells	IC_50_: 720 and 700 µM	Pancreatic, breast, colon, and melanoma	[339]
*Geranium thunbergii*	Geraniin	C_41_H_28_O_27_	952.6	↓MMP2, ↓Fak, ↓Src, ↓ERK ½	SCC-9 and SCC-14 cells	20–80 µM	Brain, breast, colon, ovarian, bladder, and osteosarcoma	[340]
*Forsythia suspensa*	**Phillygenin**	C_21_H_24_O_6_	372.4	↑Caspase-3/-9, ↑Bax, ↑ROS, ↓Bcl-2, G2/M phase arrest, ↓Nf-kβ	SH-1-V1 cell line and xenograft in mice	IC_50_: 6 µM	Lung, liver, and pancreatic cancer	[341]
*Enicosanthellum phalcrum*	Liriodenine	C_17_H_9_NO_3_	275.26	↑Bax, ↑Caspase-3, ↓Bcl-2, G2/M phase arrest	ECA-109 cell line	0.1–20 µM	Breast, prostate, and gastric cancer	[342]
*Halichondria* sp.	Ilimaquinone	C_22_H_30_O_4_	358.5	↑ROS, ↑LC3B-II, ↑Atg5, ↓p-pAkt, ↓p-p38, ↓HIF-1α, ↓Mcl-1, ↓Bcl-2	SCC4 and SCC2095 cells	IC_50_: <9 µM	Prostate, lung, and colon cancer	[343]
*Tabernaemontana catharinensis*	Coronaridine	C_21_H_26_N_2_O_2_	338.4	↑apoptosis, ↑cytotoxicity	Hep-2 cell line	IC_50_: 54.47 µg/mL	Leukemia, breast, colon, and gastric cancer	[344]
*Chelidonium majus*	Ukrain	C_66_H_75_N_6_O_18_PS	1303.4	↓EGFR, ↓AKT2, ↓STAT3, JAK1, ↓β-catenin, ↑CYP1A1, ↑CYP1B1	FaDu, HlaC78 cells	EC_50_: <11 µg/mL	Lung and prostate cancer	[345]
**Prostate Cancer**								
*Cruciferae*	Indole-3-carbinol	C_9_H_9_NO	147.17	↓CDK6, ↑Bax, ↓Bcl-2, ↑p21, ↑p27, G1 phase arrest	PC-3 cells	30–100 µM	Melanoma, colon, breast, and endometrial	[346]
*Genista tinctoria*	Genistein	C_15_H_10_O_5_	270.24	↓HOTAIR, miR-34a↓NFκβ, ↓Akt	PC3 and Du145 cells	25 µM	Lung and breast cancer	[347,348]
*Punica granatum*	Ellagic acid	C_14_H_6_O_8_	302.19	↑IL-6, ↓STAT3, ↓Akt, ↓ERK	LNCaP and PC-3 cells	20–100 µM	Breast and ovarian cancer	[349,350]
*Stephania tetrandra*	Fangchinoline	C_37_H_40_N_2_O_6_	608.7	↓NR4A1, ↓survivin, ↑ROS, ↑caspase 3 and 8	MiaPaCa-2 and Panc-1 cells	IC50: 11.1 and 17 µM	Gastric, bladder, breast, and colon cancer	[351]
*Melodinus khasianus*	**Khasuanine A**	C_21_H_24_N_2_O_3_	352.43	↑Caspase-3, ↑p53, ↓Bcl-2	PC3 cell line	IC50: 0.45 µM	Breast, lung, and colon cancer	[352]
*Camptotheca acuminate*	**Camptothecin**	C_20_H_16_N_2_O_4_	348.4	↑ROS, ↑c-Myc, ↑sp1, ↑PI3k/Akt, ↑hTERT, G2/M cycle arrest	LNCaP cells	1–5 µM	Leukemia, breast, colon, and lung cancer	[353]
*Andrographis paniculata*	Andrographolide	C_20_H_30_O_5_	350.4	↓MMP11, ↑γH2AX, ↑ Caspase-3, and 7, G2/M phase arrest	PC3, LNCaP, and 22RV1 SCID orthotopic model	GI_50_: 26.2, 28.1 and 24.2 µM	Glioblastoma, renal, colon, and ovarian cancer	[354]
*Dioscorea nipponica*	**Diosgenin**	C_27_H_42_O_3_	414.6	↓Bcl-2, ↓beclin-1, ↑caspase-9, ↓ PI3K, ↓Akt, ↓mTOR,	DU145 cell line	IC50: 6.757 µg/mL	Breast, liver, and gastric cancer	[355]
*Murraya koenigii*	Mahanine	C_23_H_25_NO_2_	347.4	↓DNMT1, ↓DNMT3B, ↓PDK1, ↓Akt, ↑RASSF1A	LNCaP and PC-3 cells	10–20 µM	Colon, brain, and lung cancer	[356]
*Quercus petraea*	Procyanidin	C_30_H_26_O_13_	594.5	↓ΔΨ_m,_ ↑ apoptosis, and necrosis	PC-3 cell line	100–300 µg/mL	Breast, lung, stomach, and colon cancer	[357]
*Santalum album*	α-santalol	C_15_H_24_O	220.35	↓Survivin, ↓p-Akt, ↑ Caspase-3, ↑cleaved PARP	LNCaP and PC-3 cells	20 and 40 µM	Breast, colon, and skin cancer	[358]
*Goniothalamus* spp.	Altholactone	C_13_H_12_O_4_	232.23	↓p65, ↓NF-kβ, ↓STAT3, ↓survivin, ↓Bcl-2, ↑Bax, S phase arrest	DU145, PC3, and LNCap cells	20 and 40 µM IC50: 38.5 µM	Colon, leukemia, and bladder cancer	[359]
*Nauclea subdita*	Subditine	C_20_H_15_N_3_O_2_	330.1237	↑ROS, ↓MMP, ↑cym-c, ↑caspase-3,7 and 9, ↓Bcl-2	LNCaP and PC-3 cells	IC50: <14 µM	Breast, lung, and colon cancer	[360]
*Isodon eriocalyx*	**Eriocalyxin B**	C_20_H_24_O_5_	344.4	↑cleaved caspase 3 and 8, ↑cleaved PARP, ↑LC3B-II, ↓Akt/mTOR	PC3 and 22RV1 cells	IC50: <4 µM	Breast, colon, lung, and bladder cancer	[361]
*Garcinia indica*	Garcinol	C_38_H_50_O_6_	602.8	↑ Bax/Bcl2, ↑caspase-3 and 9, ↓PARP, ↑PI3K, ↑Akt, ↑mTOR	PC-3 cells and xenograft mice	30 µM	Breast, lung, leukemia, and bladder cancer	[362]
*Allium atroviolaceum*	Tricin	C_17_H_14_O_7_	330.29	↓MiR-21	PC-3 cell line	IC50: 117.5 µM	Breast, colon, and liver cancer	[363]
*Sinomenium acutum*	Sinomenine	C_19_H_23_NO_4_	329.4	↓miR-23a, ↓CD1, ↓CDk4, ↓Bcl-2, ↓MMP-2 and 9, ↓PI3K/Akt, ↓JAK/STAT	PC-3 cells	0.25–1 mM	Gastric, ovarian, breast, and lung cancer	[364]
**Hematological Cancer**								
*Artemisia annua*	Dihydroartemisinin	C_15_H_24_O_5_	284.35	↓VEGF, ↓ERK1/2	RPMI18226 MM cells	IC50: 30.24 µmol/L	Brain, melanoma, and ovarian	[365]
*Artemisia absinthum*	Cardamonin	C_16_H_14_O_4_	270.28	↑ROS, ↑Ca2+, ↑ Caspase-3, -8 and-9, ↓Bcl-2, ↑Bax, ↑ cyto-c, ↑AIF, ↑GRP78, ↑FasL, ↑DAP,	WEHI-3 cell line	2–10 µM	Gastric, nasopharyngeal, prostate, and colon cancer	[366]
*Myristica fragrans*	Myristicin	C_11_H_12_O_3_	192.21	↑Caspase-3, ↑Cyto- C, ↓PARP, ↓ERCC1, ↓RAD50, 51, ↓ATM	K562 cells	IC_50_: 368 µM	Stomach, lung, and ovarian cancer	[367]
*Ganggui luhui wan*	Meisoindigo	C_17_H_12_N_2_O_2_	276.29	↑Caspase-3,8 and 9, ↑Bax, ↑Cyto- C, ↑PARP, ↓Bcl-2,	HL60 cell line	20 µmol/L	Colon, breast, and lung cancer	[368]
*Cananga odorata*	Sampangine	C_15_H_8_N_2_O	232.24	↑ROS, ↓ΔΨ_m,_ ↑Caspase-3, G1 phase arrest	HL60 cell line	1–20 µM	Lung, head, and neck cancer	[369]
*Ambrosia maritoma*	**Damsin**	C_15_H_20_O_3_	248.32	↓c-Src, ↓AKT, STAT5, ↓NFkβ	CEM/ADR5000, CCRF CEM cells	IC_50_: 4.8 µM	Colon, breast, and lung cancer	[370]
*Forsythia suspensa*	Pinoresinol	C_20_H_22_O_6_	358.4	↑p21 ^WAF1/Cip1^, G0/G1 phase arrest	HL60, HL60R, and K562 cells	IC_50_: 8 and 32 µM	Breast, prostate, and colon cancer	[371]
*Danggui longhui wan*	**Indirubins**	C_16_H_10_N_2_O_2_	262.26	↓c-Src, ↓Abl kinase, ↓Gsk3β	KCL-22 and T315I mutant KCL-22 cells	IC_50_: 0.3–0.9 µM	Colon, breast, renal, and pancreatic cancer	[372]
*Arnica chamissonis*	Helenalin	C_15_H_18_O_4_	262.3	↓NFkβ, ↑ROS	Jurkat T (J16) and Neo jurkat or Bcl-2 jurkat cells	2–20 µM	Breast, prostate, and colon cancer	[373]
*Cinnamosma fragrans*	**Capsicodendrin**	C_34_H_48_O_10_	616.7	DNA damage and proapoptotic	K562 and HL-60 cells	IC_50_: <0.5 µM	Breast, lung, and colon cancer	[374]
*Cephalotaxus fortunei*	**Homoharringtonine**	C_29_H_39_NO_9_	545.6	↓SP1/TET1/5hmC/FLT3/MYC	MA9.3ITD, MA9.3RAS, MONOMAC 6, Kasumi-1 and xenograft in mice	IC_50_: 9.2–36.7 nM	Colon, breast, and gastric cancer	[375]
*Papaver somniferum*	Noscapine	C_22_H_23_NO_7_	413.4	↓Bcl-2, ↓XIAP, ↓Mcl-1, ↓Bcl-xl, ↑Bax, ↓TRAF1, ↓IKK, ↓NFkβ,	KBM-5 and U266 cells	IC_50_: 84.4 and 155 µmol/L	Colon, breast, lung, and ovarian cancer	[376]
*Streptomyces cinnamonensis*	**Monensin**	C_36_H_62_O_11_	670.9	↓MyB, ↓MyB-NFIB	HEK-Luc, HEK-MyB-Luc, and MyB-NFIB ACC cells	0.3–3 µM	Prostate, ovarian, colon, and pancreatic cancer	[377]
*Brucea antidysenteriea*	**Bruceantin**	C_28_H_36_O_11_	548.6	↑NOTCH1, ↑HES1, G1 phase arrest	MM-CSCs cells	IC_50_: 77 nM	Breast, colon, osteosarcoma, and lung cancer	[378]
*Aglaia foveolata*	**Silvestrol**	C_34_H_38_O_13_	654.7	↓FLT3, ↓*miR155*, ↓ p65	Mv4-11, THP-1, AML blasts from patients and Mv4-11 xenograft murine model	IC_50_: 2.7, 3.8 and 12 nM	Liver, colon, and melanoma	[379]
**Miscellaneous Cancer**								
*Cynanchum otophyllum*	Paeoniflorin	C_23_H_28_O_11_	480.5	↑HTRA3, ↑Bax	Capan-1 and MIAPaCa-2 cells	IC_50_: 862.7, and 489.5 µM	Pancreatic, glioma, lung, and colon cancer	[380]
*Periploca sepium bunge*	Baohuoside-I	C_27_H_30_O_10_	514.5	↓β-catenin, ↓survivin, ↓cyclin D1	Eca109 cells and Eca109-Luc xenograft in mice	12.5–50 µg/mL IC_50_:24.8 µg/mL	Esophageal, breast, and leukemia	[381]
*Ochrosia elliptica*	Ellipticine	C_17_H_14_N_2_	246.31	↑AIF, ↑Cyto- C, ↑ROS, ↑caspase-3, 7 and 9, ↑ERK, ↑JNK, G2/M cycle arrest	RL95-2 cells	1–10 µM	Endometrial, ovarian, thyroid, and breast cancer	[382]
*Ilex hainanensis*	Ilexgenin A	C_30_H_46_O_6_	502.7	↓IL-6, ↑TNF-αG0/G1 cycle arrest	RAW 264.7 and B16-F10 xenograft in mice	IC_50_: 66.57 and 27.34 µM	Melanoma, colon, breast, and cervical cancer	[383]
*Dysoxylum binectariferum*	Flavopiridol	C_21_H_20_ClNO_5_	401.8	↑caspase-3, 8 and 9, ↓Mcl-1 ↑cyclin-B1, G2/M cycle arrest	KKU-055, -100, -214 cells and KKU-213 xenograft in Balb/c mice	IC_50_: 40–213 nM	Osteosarcoma, leukemia, lung, and breast cancer	[384]
*Crinum asiaticum*	Crinamine	C_17_H_19_NO_4_	301.34	↓Snail1, ↓vimentin, ↓VEGF-A, ↓CDK4, ↓RHOA, ↓PLK1, ↓BCL2L1, ↓Akt1	SiHa and C33a cell lines	IC_50_: 23.52 and 60.89 µM	Cervical, prostate, gastric, and colon cancer	[385]
*Alpinia katsumadai*	Alpinetin	C_16_H_14_O_4_	270.28	↓Bcl-2, ↓Bcl-xL, ↑ Bax, ↑Cyto- C, ↑XIAP, ↑caspase-3, 8 and 9	BxPC-3, PANC1, and AsPC-1 cells	20–80 µg/mL	Hepatoma, leukemia, colon, lung, and breast cancer	[386]
*Combretum caffrum*	**Combretastatin A4**	C_18_H_20_O_5_	316.3	↓N-cadherin, ↓Vimentin, ↓slug, ↓snail1, ↓zeb1, ↓p-PI3K, ↓p-Akt	TPC1 cell line	5 and 10 µM	Thyroid, breast, colon, and lung cancer	[387]
*Heliotropium Indicum Linn*	Indicine N-oxide	C_15_H_25_NO_6_	315.36	↑p53, cleave PARP, cleave DNA	Hela, MCF-7, PC3, and SiHa cell lines	IC_50_: 46–91 µM	Colon, breast, prostate, and head and oral cancer	[388]
*Arnebia euchroma*	Alkannin	C_16_H_16_O_5_	288.29	↑ROS, ↓ΔΨ_m,_ ↑p38 MAPK, ↑JNK	MDA-MB-231, HCT116, A549, Huh7, HepG2, MCF cells	IC_50_: 5–12 µM	Breast, colon, lung, liver, and glioma	[389]
*Artemisia princeps*	Jaceosidin	C_17_H_14_O_7_	330.29	↑pCdc25C, ↑p21, ↑ROS, ↓ERk ½, ↑ATM-Chk1/2, G2/M phase arrest	HES, HESC, Hec1, and KLE cell line	IC_50_: 52.68–147.14 µM	Endometrial, ovarian, glioblastoma, and oral cancer	[390]
*Coccinia grandis*	Kaempferol	C_15_H_10_O_6_	286.24	↑LC3-II, ↓p62, ↓G9a, ↑IRE1-JNK-CHOP	AGS, NCI-N87, MKN-74, SNU-216, and SNU-638	IC_50_: 50 µM	Gastric, colon, breast, prostate, and pancreatic cancer	[391]
*Daphne odora*	Daphnetin	C_9_H_6_O_4_	178.14	↓RhoA, ↓Cdc42	LM8 OS cells	1–30 µM	Osteosarcoma, breast, lung, and colon cancer	[392]
*Cerbera odollam*	**Cerberin**	C_32_H_48_O_9_	576.7	↓cMyc, ↑Caspase 3 and 7, ↑ROS ↓Bcl-2, ↓Mcl-1, ↓STAT-3, ↓PLK-1, G2/M phase arrest	PANC-1, MIA Paca2, A549, HepG2, HT29 cells and mice model	GI_50_: <90 nM	Pancreatic, liver, colon, and breast cancer	[393]
*Asiatic toad*	**Cinobufagin**	C_26_H_34_O_6_	442.5	↓Bcl-2, ↓CB1, ↑p21, ↓CDC2, ↑puma, ↑caspase 3, G2/M phase arrest	EC-109, Kyse-150, and Kyse-520 cells	IC_50_: 0.91, 0.66 and 0.62 µM	Gastric, liver, colon, and melanoma	[394]
*Astragalus membranaceus*	Swainsonine	C_8_H_15_NO_3_	173.21	↑E-cadherin,↓N-cadherin, ↓Vimentin, ↓slug, ↓snail1, ↓zeb1, ↓p-PI3K, ↓p-Akt, ↓Twist1	EC9706 and 293T cells	10–100 µg/mL	Esophageal, colon, liver, and glioma	[395]
*Magnolia officinalis*	Honokiol	C_18_H_18_O_2_	266.3	↓cyclin D1, ↓CDK2 and 4, ↓Akt/mTOR, ↓p38, ↓ERK, G0/G1 phase arrest	WRO, SW579 cells, and ARO cells xenograft in Balb/c mice	IC_50_: 37.7, 19.9, and 36.3 µM	Thyroid, leukemia, prostate, and colon cancer	[396]
*Polymethoxyflavone*	Tangeritin	C_20_H_20_O_7_	372.4	↓ MMP, ↑ Caspase-3, -8 and -9, ↑ Bax, ↑ Bid, ↑ tBid, ↑ p53, ↑ p21/cip1, ↑ Fas and ↑ FasL	AGS cell line	5–240 µM	Gastric, colon, and lung cancer	[397]
*Amoora rohituka*	Aphanin	C_35_H_54_O_4_	538.80	↓K-Ras, ↓p-AKT, ↓cMyc, ↓cyclin D1, ↓STAT3, G0/G1 cycle arrest	HPAF-II, BxPC3, and HPAC cells	IC_50_: 12.80, 15.68 and 17.26 µM	Pancreatic, lung, colon, liver, and skin cancer	[398]
*Eucalyptus globulus*	Caffeic acid	C_9_H_8_O_4_	180.16	↑ Caspase-1, 3, and 8, G0/G1 cycle arrest	SK-Mel-28 cells	25–200 µM	Melanoma, breast, colon, gastric, and ovarian cancer	[399]
*Zingiber officinale*	6-gingerol	C_17_H_26_O_4_	294.4	↑ROS, ↑Bax/Bcl-2, ↑Caspase-3, 9, ↑cym-c, G2/M phase arrest	AGS cell line	IC_50_: 250 µM	Liver, lung, breast, and retinoblastoma	[400]
*Humulus lupulus*	Xanthohumol	C_21_H_22_O_5_	354.4	↑caspase-3, 8 and 9, ↑PARP, ↑p53, ↑AIF, ↓Bcl2, ↓XIAP, S phase cycle arrest	Ca Ski cell line	IC_50_: 20 µM	Cervical, breast, and mammary adenocarcinoma	[401]
*Trollius chinensis*	Orientin and Vitexin	C_21_H_20_O_11_, C_21_H_20_O_10_	448.4, 432.4	↑p53, and ↓Bcl2	EC-109 cells	5–80 µM	Esophageal, breast, colon, and lung cancer	[402]
*Rhodiola rosea*	Salidroside	C_14_H_20_O_7_	300.3	↓p-MAPK, ↓p-ERK, ↓p-PI3K, ↓p-AKT, ↓miR99a, ↑p21, ↓Bcl-2	GES-1, NU-216 and MGC803 cells	0.8–8000 µM	Gastric and bladder cancer	[403]
*Syzgium aromaticum*	Eugenol	C_10_H_12_O_2_	164.2	↑ROS, ↑Bax, ↓PCNA, ↓ΔΨ_m,_ G2/M cycle arrest	SIHA, SK-MEL-28, A2058, MCF-7, MDA-MB-231	IC_50_: <23 µM	Cervical, breast, colon, and lung cancer	[404]
*Uapaca togoensis*	Arborinine	C_16_H_15_NO_4_	285.29	↓MMP, ↑ROS, G0/G1 and S phase arrest	CCRF-SEM, MDA-MB-231, HCT119, U87 MG, HepG2 cells	IC_50_: <10 µM	Leukemia, brain, breast, and pancreatic cancer	[405]
*Peganum harmala*	Harmine	C_13_H_12_N_2_O	212.25	↑beclin-1, ↑LC3-II, ↓Bcl-2, ↑Bax, ↓Akt/mTOR, ↓p70S6k	MGC-803 and SGC-7901 cells	IC_50_: <59 µM	Gastric, breast, ovarian, and pancreatic cancer	[406]
*Artemisia vestita*	Hispidulin	C_16_H_12_O_6_	300.26	↓VEGFR2, ↓Akt, ↓mTOR, ↓S6 kinase, ↓PI3K, ↓Bcl-2	PANC-28, BxPC-3, HUVEC, PANC-1 xenograft BALB/c mice	IC_50_: 20–200 µmol/L	Pancreatic, glioblastoma, and ovarian cancer	[407]
*Didemnum cuculiferum*	**Vitilevuamide**	C_77_H_114_N_14_O_21_S	1603.9	↓tubulin polymerization, G2/M phase arrest	A498, HCT116, A5249, and SK-MEL-5 cells and p388 xenograft in mice	LC_50_: 6–311 nM	Kidney, colon, prostate, ovarian, and pancreatic cancer	[408]
*Piper longum*	Piperlongumine	C_17_H_19_NO_5_	317.34	↑p-elF2α, ↓ΔΨ_m_, ↑ATF4, ↑CHOP, ↓TrxR1	SGC-7901, BGC-823, KATO III cell lines	0.625–20 µM	Gastric, breast, lung, and colon cancer	[409]
*Chrysanthemum parthenium*	Parthenolide	C_15_H_20_O_3_	248.32	↓NFκβ, ↓VEGF, ↓c-jun, ↓c-Fos	KYSE510, Het-1A, and Eca109 xenograft BALB/c mice	IC_50_: 13.3, 21.54, and 10.3 µM	Esophageal carcinoma, colon, and prostate cancer	[410]
*Solanum nigrum* L.	Degalactotigonin	C_50_H_82_O_22_	1035.2	↓Hedgehog Gli1, ↓GSK3β	U2OS, U2OS/MTX, ZOS-M, and ZOS xenograft in mice	IC_50_: 12.91 –31.46 µmol/L	Osteosarcoma, colon, and pancreatic cancer	[411]

**Note:** Bold font chemical compounds represent the compounds with good anticancer potential against the selected cancer model (lower IC_50_ values).

**Table 2:** Natural products undergoing clinical trials for the prevention/treatment of various cancers.

Cancer	Bioactives	Class/Family	Combination With Other Drugs	Clinical trial Status	Results	Purpose	References
Lung	Curcumin	Phenol	Gefitinib/Erlotinib	Unknown	Not reported	Safety and Tolerability	NCT02321293 *
	-	-	Lovaza	Ongoing	-	Prevention	NCT03598309 *
	Epigallocatechin	Catechin	mLDG	-	-	Treatment	NCT02577393 *
	Sulforaphane	Organosulfur		-	-	Prevention	NCT03232138 *
	Gossypol	Phenolic aldehyde	DTX, CIS	Unknown	Not reported	Treatment	NCT01977209*
Breast	ATRA	Retinoid	Anastrozole	Ongoing	-	-	NCT04113863 *
	Curcumin	Phenol		-	-	-	NCT03980509 *
	-	-	Paclitaxel	Completed	Not posted	-	NCT03072992 *
	Genistein	Isoflavone	Gemcitabine	-	Efficacious	Treatment/Prevention	NCT00244933 *
	7-hydroxystaurosporine	Staurosporine derivative		-	Not posted	Treatment	NCT00001444 *
	Emodin	Resin		Unknown	Not posted	Observational	NCT01287468 *
	Cognutrin	Fatty acid + Phenols		Completed	Efficacious	Treatment	NCT01823991 *
Cervical/Ovarian	Curcumin	Phenol	Cyclophosphamide, Lansoprazole, Aspirin, Vitamin D, Pembrolizumab	Ongoing	-	-	NCT03192059 *
	OPT-821	Saponin	Antigen-KLH conjugate Vaccine	Completed	Efficacious	Treatment	NCT00857545 *
Colon	Curcumin	Phenol	5-FU	Ongoing	-	-	NCT02724202 *
	-	-	Irinotecan	Completed	Not posted	Toxicity and Pharmacokinetics	NCT01859858 *
	-	-	Avastin/FOLFIRI	Completed	Not reported	Treatment	NCT02439385 *
	-	-	FOLFOX	Completed	-	-	NCT01490996 *
	-	-	Celecoxib	Unknown	-	-	NCT00295035 *
	Andrographolide	Diterenoid	Capecitabine	Terminated	Low accrual rate	Treatment	NCT01993472 *
	Berberine	Benzylisoquinoline alkaloid		Ongoing	Not posted	Prevention	NCT03281096 *
	Aquamin	Multi-mineral complex	Calcium carbonate	Active	-	-	NCT02647671 *
	Cyanidin-3-glucoside	Anthocyanin	Curcumin	Unknown	-	Prevention	NCT01948661 *
	Ellagic Acid	Phenol		Completed	-	Treatment	NCT01916239 *
	Silymarin	Flavonolignan		Completed	Unknown	-	NCT03130634 *
Brain	Chlorogenic acid	Phenol		-	Not posted	Treatment	NCT02728349 *
	Perillyl alcohol	Terpenes		Ongoing	-	Treatment	NCT02704858 *
Head and Neck	Combretastatin A4 Phosphate	Phenol derivative		Completed	-	Treatment	NCT00060242 *
	β-Carotene	Terpenoid	α-Tocopherol	-	-	Prevention	NCT00169845 *
	Capsaicin	Capsaicinoid	Radiation therapy	-	-	Supportive care	NCT00003610 *
Liver	Silybin	Flavonoid		-	-	Treatment	NCT01129570 *
Prostate	Curcumin	Phenol		-	-	Supportive	NCT01917890 *
	-	-	Taxotere	Terminated	No Significant outcome	-	NCT02095717 *
	Lycopene	Carotenoid	Vit D3 and E, Selenium, green tea extract	Completed	Not posted	Treatment	NCT00844792 *
	Polyphenon E	polyphenol	EGCG	-	Efficacious	Treatment/prevention	NCT00676780 *
	Cholecalciferol	Vitamin D		-	Effective	-	NCT00524680 *
Hematological	Bioperine	Alkaloid	Curcumin	-	-	-	NCT00113841 *
	Plitidepsin	Depsipeptide	Bortezomib and dexamethasone	-	Not posted	-	NCT02100657 *
	Bryostatin 1	polyketide		-	-	-	NCT00003171 *
	Homoharringtonine	Alkaloid		-	-	-	NCT00006364 *
Skin cancer	Ingenol Mebutate	Diterpene ester		-	-	-	NCT01325688 *
Advanced solid tumors	Elisidepsin	Depsipeptide	Erlotinib	-	Not posted	-	NCT00884845 *
	Discodermolide	Polyketide		-	unknown	-	[429]
Tongue	Luteolin	Flavonoid		Unknown	Not posted	Treatment	NCT03288298 *
GI Tumors	Resveratrol	Phenol		Completed	Not posted	Treatment	NCT01476592 *
Pancreatic	Etoposide	Podophyllotoxin derivative	Gemcitabine	-	-	-	NCT00202800 *

* represent the clinical trial number obtained from https://clinicaltrials.gov (accessed on 1 June 2022).

## Data Availability

Not applicable.
